# Genome-wide analysis of long noncoding RNAs and their association in regulating the metamorphosis of the *Sarcophaga peregrina* (Diptera: Sarcophagidae)

**DOI:** 10.1371/journal.pntd.0011411

**Published:** 2023-06-26

**Authors:** Yanjie Shang, Yakai Feng, Lipin Ren, Xiangyan Zhang, Fengqin Yang, Changquan Zhang, Yadong Guo

**Affiliations:** 1 Department of Forensic Science, School of Basic Medical Sciences, Central South University, Changsha, Hunan, China; 2 Department of Forensic Science, School of Basic Medical Sciences, Xinjiang Medical University Ürümqi, China; Kenya Agricultural and Livestock Research Organization, KENYA

## Abstract

**Background:**

The flesh fly, *Sarcophaga peregrina* (Diptera: Sarcophagidae), is an important hygiene pest, that causes myiasis in humans and other mammals, typically livestock, and as a vector for various parasitic agents, including bacteria, viruses, and parasites. The role of long non-coding RNAs (lncRNAs) in regulating gene expression during metamorphosis of the flesh fly has not been well established.

**Methodology/Principal findings:**

In this study, we performed genome-wide identification and characterization of lncRNAs from the early pupal stage (1-days pupae), mid-term pupal stage (5-days pupae), and late pupal stage (9-days pupae) of *S*. *peregrina* by RNA-seq, and a total of 6921 lncRNAs transcripts were identified. RT-qPCR and enrichment analyses revealed the differentially expressed lncRNAs (DE lncRNAs) that might be associated with insect metamorphosis development. Furthermore, functional analysis revealed that the DE lncRNA (*SP_lnc5000*) could potentially be involved in regulating the metamorphosis of *S*. *peregrina*. RNA interference of *SP_lnc5000* caused reduced expression of metamorphosis-related genes in 20-hydroxyecdysone (20E) signaling (Br-c, Ftz-F1), cuticle tanning pathway (TH, DOPA), and chitin related pathway (Cht5). Injection of ds*SP_lnc5000* in 3rd instar larvae of *S*. *peregrina* resulted in deformed pupae, stagnation of pupal-adult metamorphosis, and a decrease in development time of pupal, pupariation rates and eclosion rates. Hematoxylin-eosin staining (H&E), scanning electron microscope (SEM) observation and cuticle hydrocarbons (CHCs) analysis indicated that *SP_lnc5000* had crucial roles in the metamorphosis developmental by modulating pupal cuticular development.

**Conclusions/Significance:**

We established that the lncRNA *SP_lnc5000* potentially regulates the metamorphosis of *S*. *peregrina* by putatively affecting the structure and composition of the pupal cuticle. This study enhances our understanding of lncRNAs as regulators of metamorphosis in *S*. *peregrina*, and provide valuable insights into the identification of potential targets for vector control and the development of effective strategies for controlling the spread of myiasis and parasitic diseases.

## Author summary

*Sarcophaga peregrina*, a significant flesh fly species, is closely associated with human life in its ecological habits, as vectors of parasitic disease agents, they can cause myiasis and other tropical infectious diseases, and has been implicated in the transmission of diseases such as cutaneous leishmaniasis. Enhance the understanding of metamorphosis of this species, have a significance for vector control. The lncRNAs plays important roles in growth development of insect. However, its role in regulating the metamorphosis of *S*. *peregrina* has not been reported so far. In this study, we obtained genome-wide identification of lncRNA profiles from different pupae developmental stages of *S*. *peregrina*. Furthermore, we performed a functional analysis of differentially expressed lncRNAs (*SP_lnc5000*) in regulating the metamorphosis of *S*. *peregrina* by RNA interference, and H&E staining, SEM observation and CHCs detection. The results provided here increase the knowledge on metamorphosis processes in *S*. *peregrina*, which is important for the design of new vector control strategies.

## Introduction

Noncoding RNAs, such as long noncoding RNAs (lncRNAs), small interfering RNAs, piwi-interacting RNAs, circular RNAs and microRNAs, play key regulatory functions in a variety of physiological processes including development, immunity, apoptosis and host-microbe interactions [1]. Among these noncoding RNAs, lncRNAs are a type of nonprotein-coding RNA with a length of more than 200 nucleotides that have been detected in a variety of animals [2]. RNA polymerase II produces lncRNAs, which go through polyadenylation, capping, and alternative splicing just like protein-coding mRNAs [3]. LncRNAs are classified into four groups based on the position and direction of transcription in relation to protein-coding genes: sense, antisense, intronic, and intergenic [4].

LncRNAs have been found to play key roles in growth, development, innate immunity, gene expression regulation, and other biological processes in a number of studies [5]. A high percentage of lncRNA expression is up-regulated in *Drosophila melanogaster* late embryonic and larvae stages, suggesting that lncRNAs have key regulatory functions in metamorphosis [6]. A phylogenetic analysis revealed that lncRNAs are less conserved even among evolutionarily related species [7]. Identification of lncRNAs in various insects is required. At present, lncRNAs have been discovered in insects such as the *Anopheles gambiae* [8], *Bombyx mori* [9], *Aedes aegypti* [10], *Plutella xylostella* [11], *Apis cerana*, *Apis mellife* [12] and *Sogatella furcifera* [13] by RNA-seq. However, these studies only provide a limited amount of information about lncRNAs in insects, and exploration in a wide range of species and further functional analysis is necessary.

Insect metamorphosis is a complex process, particularly in holometabolous insects, whose growth and development must go through a series of discrete stages (larvae, pupae, adult) [14]. During the process of metamorphosis, larvae tissues must go through a series of developmental events and undergo programmed cell death to remodel insect architecture through cell proliferation and differentiation [15]. The larvae bear no resemblance to their adult parents, and the transition from larva to adult requires a significant amount of morphogenesis, as well as an intermediate stage known as the pupal stage [16]. During the pupal stage, tissues and organs begin to form, cells proliferate rapidly, differentiated cells with defined functions develop, cell polarity occurs, and cytoskeletal remodeling and various essential adult tissues and organs emerge [17,18]. Therefore, understanding the regulatory mechanisms of evolution and growth of insects requires a comprehensive understanding of the metamorphic development of the pupal.

*Sarcophaga peregrina* (Robineau-Desvoidy, 1830) (Diptera: Sarcophagidae), is an important hygiene pest, and as vectors of parasitic disease agents, they can cause myiasis in humans and other mammals [19]. Several human myiasis cases caused by *S*. *peregrina* have occurred in different sites, such as the nose, mouth and the eyes, suggesting that proper control measures for flies involved in myiasis in humans are necessary [20]. In addition, *S*. *peregrina* is known to act as a vector for various parasitic agents, including bacteria, viruses, and parasites, and has been implicated in the transmission of diseases such as cutaneous leishmaniasis [21]. Of note, Shey-Njila et al conducted an epidemiological survey of canine leishmaniasis in Cameroon, and found evidence suggesting that *S*. *peregrina* may be involved in the transmission of the disease [22]. *S*. *peregrina* is a typical holometabolous diptera insect that must go through larvae-pupal metamorphosis to molt into an adult, with the pupal stage lasting up to 50% of the whole growth and even several weeks [23]. Pupae metamorphosis is a complex developmental process that involves significant physiological and anatomical changes [24]. Studying the regulation mechanism of *S*. *peregrina* metamorphosis can provide more molecular targets for pest control. Although metamorphosis plays important roles in the evolutionary success of insects, its regulatory mechanisms are still not completely understood, and the relationship between lncRNA and the metamorphosis developmental of flesh flies is unknown [25]. RNA-sequencing is often used to study the lncRNA of insects [26].

In the present study, we aimed to better understand the regulatory mechanism of lncRNAs in *S*. *peregrina* metamorphosis, and to develop effective strategies for vector control. Firstly, we performed a genome-wide identification and characterization of lncRNAs from the early pupal stage (1-day pupae), mid-term pupal stage (5-day pupae), and late pupal stage (9-day pupae) of *S*. *peregrina* by RNA-seq. Furthermore, we validated differentially expressed lncRNAs by RT-qPCR and performed functional analysis of lncRNAs. Subsequently, we investigated the roles of *SP_lnc5000* in regulating the metamorphosis development of *S*. *peregrina* through related RNAi and H&E staining, SEM observation, and cuticle hydrocarbons (CHCs) analysis. This study adds to our knowledge of lncRNA-mediated metamorphosis development and physiological processes in *S*. *peregrina*, and more importantly, provides new strategies and molecular targets for controlling the spread of myiasis and parasitic diseases.

## Methods

### Insect rearing and sample collection

Adult specimens of *S*. *peregrina* were collected in 2020 in Hunan Province, China and reared in an artificial climate chamber at 25 ± 0.5°C, 75 ± 2% relative humidity, and with a 12h:12h light:dark cycle. The *S*. *peregrina* were reared and maintained as has been previously described [27]. 10-cm culture dishes containing 20g of fresh pig lung were placed in the rearing cages to induce larviposition. The larvae generated within 1 h were cultured and placed in a rearing box (17 × 12 × 8 cm) with sawdust at the bottom. The rearing boxes were put in a climate box (LRH-250-GSI, Taihong Co., Ltd, Shaoguan, China) at constant temperatures of 25°C with 75% humidity and a photoperiod of 12h:12h (L:D). Fresh pig lung was supplied regularly *ad libitum* to meet growth needs of the larvae until pupation. According to the results of morphological observation of pre-experiment (S1 Fig), the samples were collected from the stages with the largest morphological changes, including the early pupal stage P1 (1 day pupae), mid-term pupal stage P5 (5 days pupae), and later pupal stage P9 (9 days pupae). The first sampling was collected when approximately 50% of the post-feeding larvae entered the pupal stage (1 day pupae), which was set to “zero time” (control samples), then, the pupae samples were collected at 5 days and 9 days pupal developmental stage (test samples). A total of 20 pupae were randomly collected from each rearing box every sampling, and the samples were placed into a 5mL cryovial and immediately frozen in liquid nitrogen and stored at -80°C for the subsequent RNA isolation. Three biological replications of each treatment were collected for all samples.

### RNA isolation, cDNA library preparation and sequencing

The total RNA were isolated using the Trizol Total RNA Purification Kit I (MultiSciences (Lianke) Biotech Co., Ltd. Zhejiang, China) following the manufacturer’s protocol. RNA quantity and quality were assessed using the NanoDrop 2000 (Thermo, Waltham, MA, United States). RNA integrity was evaluated using the Agilent 2100 Bioanalyzer (Agilent Technologies, Santa Clara, CA, USA). The ribosomal RNA was digested from the total RNA using the Ribo-Zero Gold kit (Illumina, USA). The samples with RNA Integrity Number (RIN) ≥ 7 were subjected to the subsequent analysis. The libraries were constructed using TruSeq Stranded Total RNA with Ribo-Zero Gold according to the manufacturer’s instructions. Then these prepared libraries were sequenced on an Illumina sequencing platform and 150 bp paired-end reads were generated. All sequencing programs were performed by OE Biotech Co., Ltd (Shanghai, China).

### Identification and differential expression analysis of lncRNAs

The Trimmomatic v. 0.39 was used to filter out low-quality bases that contained adapters or poly-Ns and to produce high-quality, clean reads [28]. All clean reads were aligned to the *S*. *peregrina* reference genome published by our research group [29] (NCBI accession no. JABZEU000000000) using spliced read aligner HISAT v. 2.1.0 [30], and all transcripts were assembled via StringTie v. 2.1.3 software [31], and the new transcript was spliced. Then comparing the gene annotation information of the reference sequence obtained by Cuffcompare software, the potential lncRNA transcripts were chosen [32]. Finally, according to the characteristics of lncRNA, the candidate lncRNAs were filtered out using CPC [33], CNCI [34], Pfam [35] and PLEK [36].

The expression frequency of each transcript in each sample was calculated using Bowtie2 and Express software, and the fragments per kilobase of exon per million fragments mapped (FPKM), fold change (FC) and P-value were calculated [37]. DESeq2 software was employed to identify the DE transcripts in samples of different groups [38]. The significance of the variations in read numbers was determined using a negative binomial distribution test [39]. |Log2FC|>1 and q <0.05 of lncRNAs and mRNAs were considered to be differentially expressed transcripts. Based on expression level during developmental stages, lncRNAs were clustered by time_series with STEM (Short Time-series Expression Miner) software [40].

### Functional enrichment analysis and Co-Expression network construction

LncRNA cannot code protein, but it can regulate expression of the related mRNA [41]. We predicted the potential function of differentially expressed lncRNA with a search of adjacent genes within the upstream/downstream range of 100kb of lncRNA [42]. All the predicted target genes of lncRNAs were used to analyze Gene Ontology (GO) and the Kyoto Encyclopedia of Genes and Genomes (KEGG) pathway. Gene function annotations were performed based on the following databases: Nr (NCBI non-redundant protein sequences); Swiss-Prot (Amanually annotated and reviewed protein sequence database). GO and KEGG enrichment analyses were performed on the significantly differential expressed lncRNAs and mRNAs in each comparison by the DAVID gene annotation tool (http://david.abcc.ncifcrf.gov/). The GO terms and KEGG pathways with p < 0.05 were considered significantly enriched DE genes.

Based on the ceRNA (competing endogenous RNA) hypothesis, we established the co-expression network between differentially expressed lncRNA (DE-lncRNA), DE-miRNA and DE-mRNA. We first calculated the miRNA-lncRNA co-expression relationships, and then predicted their regulatory relationships to improve the reliability of our screening results. We used the ceRNA MuTATE method [43] to score the ceRNA relationships and applied the hypergeometric distribution algorithm to calculate the probability of shared miRNAs among these ceRNA pairs. Finally, we obtained ceRNA pairs with high reliability. The correlation p-value and Pearson’s correlation coefficient (PCC) statistic method was used to evaluate lncRNA-mRNA, lncRNA-miRNA-mRNA combination. A PCC value >0.99 and *p* value < 0.05 was considered statistically significant, and was retained for network construction using Cytoscape (version 3.4.0) [44].

### Real-Time Quantitative Polymerase Chain Reaction (RT-qPCR) analysis

The developmental expression patterns of *SP_lnc5000* during each day in the pupal stage (days 1–9) of *S*. *peregrina* were examined by RT-qPCR. The validation of DE-lncRNAs and DE-mRNAs in the early pupal stage P1 (1 day pupae), mid-term pupal stage P5 (5 days pupae), and later pupal stage P9 (9 days pupae) by RT-qPCR.

Total RNA from pupae were isolated using the Trizol Total RNA Purification Kit I (MultiSciences (Lianke) Biotech Co., Ltd. Zhejiang, China) according to the manufacturer’s instructions. The first strand of cDNA was synthesized from Total RNA (10ng-5μg) using the Evo M-MLV RT Mix Kit with gDNA Clean for qPCR (Accurate Biotechnology (Hunan) Co., Ltd) following the manufacturer’s instructions. RT-qPCR was performed using the SYBR Green Premix Pro Taq HS qPCR Kit (ROX Plus) (Accurate Biotechnology (Hunan) Co., Ltd) following the manufacturer’s instructions on an ABI 7500 Real-Time PCR system (Applied Biosystem, Carlsbad, CA, USA). Primers for the mRNAs, lncRNAs and internal control genes were designed using Primer 5.0 software (Biosoft Premier, Palo Alto, California, USA). Using *β-actin* gene as the reference gene to normalize the lncRNAs and mRNA expression level, the relative expression levels of mRNAs and lncRNAs were quantified using the 2^-△△Ct^ method[27]. The Log2FC was used to normalize the values of the DEGs. All primers are listed in Table 1.

**Table 1:** Primers used in the RNAi and RT-qPCR analysis.

RNAi	Gene name		Primer sequences (5’→3’)
	dsSP_lnc5000	Forward	TAATACGACTCACTATAGGGAGGGATATGCCAATCAACCA
		Reverse	TAATACGACTCACTATAGGGGCAAAAACTCAAGCAATTTGG
	dsGFP	Forward	TAATACGACTCACTATAGGGCTACCTGTTCCATGGCCAAC
		Reverse	TAATACGACTCACTATAGGGTTTTCGTTGGGATCTTTGGA
RT-qPCR	SP_lnc5000	Forward	TCTACCACATTTTGTTGCATGAA
		Reverse	ACATGAGAATGGTTTGTTGGGT
	β-actin	Forward	AAGAACAAGGTGAAGAGGGAC
		Reverse	TCGTCTTCATCGCGGAATATG
	TH	Forward	GGCTTATGGTGCTGGTCTCTTGTC
		Reverse	GATAGGGTTGTACGGCTGTGGAAG
	DOPA	Forward	TGCTAGTCCAGCTTGCACAGAATTG
		Reverse	CTTGTATAACACCACCGCCCTTACC
	ftz-f1	Forward	GCACCACTACCAATACCATCACCTC
		Reverse	TTGAAGAGGAGCAGGACGAGGAG
	Br-C	Forward	ATTGGCGGTGGTAATGGTAATGGTC
		Reverse	GTGTCAGTGGCGATGGCGTAAG
	Cht5	Forward	GGCACTAAAACGCAAACATGTCCAC
		Reverse	CGCCATTGTCCTGCTCCAAGTC
	evm.model.Contig10.332	Forward	CCAAAGAAAACCGATTGTCCG
		Reverse	TTTAATGGGCACAGGACGATAG
	evm.model.Contig130.34	Forward	ACAAAGCCTCTGATCCCATTC
		Reverse	GGCAACACGTTCCAATTCAG
	evm.model.Contig22.350	Forward	CTATGGAGGGTGGATTTGATGG
		Reverse	CACCTCCCCAAGCAATGTAG
	evm.model.Contig34.18	Forward	CTATGGAGGGTGGATTTGATGG
		Reverse	CACCTCCCCAAGCAATGTAG
	evm.model.Contig7.39	Forward	ACCATCGATCAATGCTACACTG
		Reverse	TCATTGTCTACCATGCGATCG
	evm.model.Contig37.176	Forward	ACAATCCCGACATCCCATTC
		Reverse	AACCATACTGACGTTGAGCC
	TCONS_00035151	Forward	AGCTTGCGTAGGACCAGGAAGG
		Reverse	CGACTCACATGCACGACCACTC
	TCONS_00035148	Forward	AGCTTGCGTAGGACCAGGAAGG
		Reverse	CGACTCACATGCACGACCACTC
	TCONS_00032543	Forward	AGTCTGCATTGCTTTGGCCTCTC
		Reverse	TTCATTCCGCCTGAGTTATGACGAC
	TCONS_00023118	Forward	ACATTTTGAACGCATATCGCAGTCC
		Reverse	ACCCTCAACCATATGTAGTCCAAGC
	TCONS_00010929	Forward	TCGCTGCAACATGAGGCTAACAAG
		Reverse	CGACAGCAACCTGGTGGACATAAG
	TCONS_00001066	Forward	GCGACAAATGTGACTGTGGCA
		Reverse	ACACTCGCCATGATGTCCTTCT

The RT-qPCR data were statistically analyzed by Prism software version 5.02 (GraphPad Software Inc. La Jolla, CA, USA) and assessed for normality or homogeneity of variance. The results are shown as the mean ± standard deviation (SD) of three biological replicates.

### RNAi

RNA interference was performed to further study biological functions of the DE lncRNA (*SP_lnc5000*) gene in *S*. *peregrina*. Primers for sequence-specific double-stranded RNA (dsRNA) synthesis were designed in ERNAi (http://www.dkfz.de/signaling/e-rnai3/idseq.php) based on the cDNA sequence of the *SP_lnc5000* gene. dsGFP was used for negative RNAi controls. The primers are listed in Table 1. PCR reactions and Sanger sequencing were performed to determine the specificity of the primers. The dsRNA of *SP_lnc5000* and GFP was synthesized in vitro according to the manufacturer’s instructions of T7 Express RNAi System (Promega, Madison, WI, USA). Amplicons were verified with DNA sequencing and the integrity of dsRNA product was confirmed with electrophoresis on a 1% agarose gel. The synthesized ds*SP_lnc5000* and dsGFP were dissolved in appropriate volumes of nuclease-free water, and the concentration was determined and adjusted to 2.0 μg/μl using a NanoDrop 2000 spectrophotometer (Thermo Fisher Scientific, Waltham, MA, USA). Samples were then stored in a refrigerator at –80°C until use.

Approximately 2 μl (4 μg) of ds*SP_lnc5000* or dsGFP were injected into the dorsal side of 2nd and 3rd abdominal segments of 3rd instar larvae (5 days larvae at 25°C) of *S*. *peregrina* by using a Drummond digital microdispenser (Drummond Scientific Co., Broomall, USA) [45]. The samples of ds*SP_lnc5000*-injected and the dsGFP controls were placed at the constant temperature of 25°C as described above, and the phenotypic changes were observed, mainly including the pupariation time, pupariation rates, the development time of pupal stage and eclosion rates. At 24 h and 48 h post injection, three-five pupae samples were collected and RT-qPCR was performed to calculate RNAi efficiency of the *SP_lnc5000* gene, and expression level variation of related genes of 20-hydroxyecdysone (20E) signaling (Br-c, Ftz-F1), cuticle tanning pathway (TH, DOPA), and chitin related pathway (Cht5) using the same methods as described above. All of the data were statistically analyzed by independent sample student t-test. Asterisks indicate significant differences (*, P < 0.05; **, P < 0.01; ***, P < 0.001). There were 30 larvae in each treatment group, and experiments were repeated independently three times.

### Scanning Electron Microscope (SEM) observations

In addition, scanning electron microscopy (SEM) was used to further compare the morphological differences of pupal cuticle between ds*SP_lnc5000*-injected group and the dsGFP control group. The pupae in later stages were cleaned in ultrapure water and allowed to dry at room temperature. Pupal cuticle was then observed under a Schottky Field Emission Scanning Electron Microscope (Hitachi, SU5000, Tokyo, Japan).

### Haematoxylin and Eosin (H&E) staining

To further observe the effects of silencing of *SP_lnc5000* gene on the pupal metamorphosis development of *S*. *peregrina*, H&E staining was performed. At later pupal stage after dsGFP- or ds*SP_lnc5000*-injection, three-five pupae samples were collected for the H&E staining. Under a Zeiss 2000-C stereomicroscope (Carl Zeiss, Germany), the puparium were taken out with ophthalmic forceps, ophthalmic scissors and insect pins, and the external morphological changes of the intra-puparial were observed and recorded [46]. The intra-puparial specimens were immersed in a formaldehyde solution for 48h, and then transferred to 70% alcohol for an additional 48h to fix the tissue. The usual procedure was followed for dehydration, paraffin infiltration, embedding, sectioning, and H&E staining [47]. The internal histological changes of the pupae after RNAi were observed and recorded using an automatic digital slice scanning and application system, as well as Motic DSAssistant Lite (Motic, USA).

### Cuticular Hydrocarbons (CHCs) analysis

At later pupal stage after *dsGFP*- or *dsSP_lnc5000*-injection, three-five pupae samples were collected for the CHCs profile analysis. The pupae was cleaned in ultrapure water and blotted dry with filter paper. Then, these pupae was immersed in 1 mL hexane in a 2mL glass vial at room temperature for an hour. Next, a syringe filter transferred the immersed liquid with a 0.45μm aperture nylon membrane. After that, the liquid was dried under vacuum and then dissolved in 100μL hexane for gas chromatography-mass spectrometry (GC-MS) analysis. GC-MS (Agilent Technologies, 7890B-5977A GC/MSD), with a DB-5MS capillary column (30m×0.25mm ×0.25μm), was used for the CHCs analysis. Equipment operation procedures referred to previous research [48]. The n-alkanes mix from heptane to tetracontane (C7-C40, 1μg/mL, O2SI) resolved in 1 mL hexane was used as an external standard. MSD ChemStation Data Analysis F.01.03 was used to integrate the peak height, and only compounds with a consistent peak height percentage above 0.5% were included. Hydrocarbons were identified using a library search (NIST14), the Kovats Index based on external standards, and literature [49].

## Results

### Characterization of the pupae tissue transcriptome

The pupal stage lasted 9 days at 25°C. Morphological characteristics of *S*. *peregrina* pupae in the early pupal stage P1 (1 days pupae), mid-term pupal stage P5 (5 days pupae), and later pupal stage P9 (9 days pupae) at 25°C are shown in S1 Fig. The *S*. *peregrina* samples from three pupal developmental stages, including the early pupal stage (P1), mid-term pupal stage (P5), and later pupal stage (P9) were analyzed by RNA-seq, and three replicates were established for each developmental stage. Approximately 79.48Mb of raw reads per sample were obtained from the 9 cDNA libraries. After removing adaptor sequences and low-quality reads, approximately 78.12Mb of clean reads were obtained from each sample. The Q30 base distribution ranged from 92.66~93.2%, and the average GC content was 39.08% (S1 Table). Finally, an average 81.17% of clean reads were successfully mapped to the *S*. *peregrina* reference genome, and approximately 73.93% of clean reads were aligned with unique loci (S2 Table).

### Identification of lncRNAs in *S*. *peregrina*

**Fig 1:**
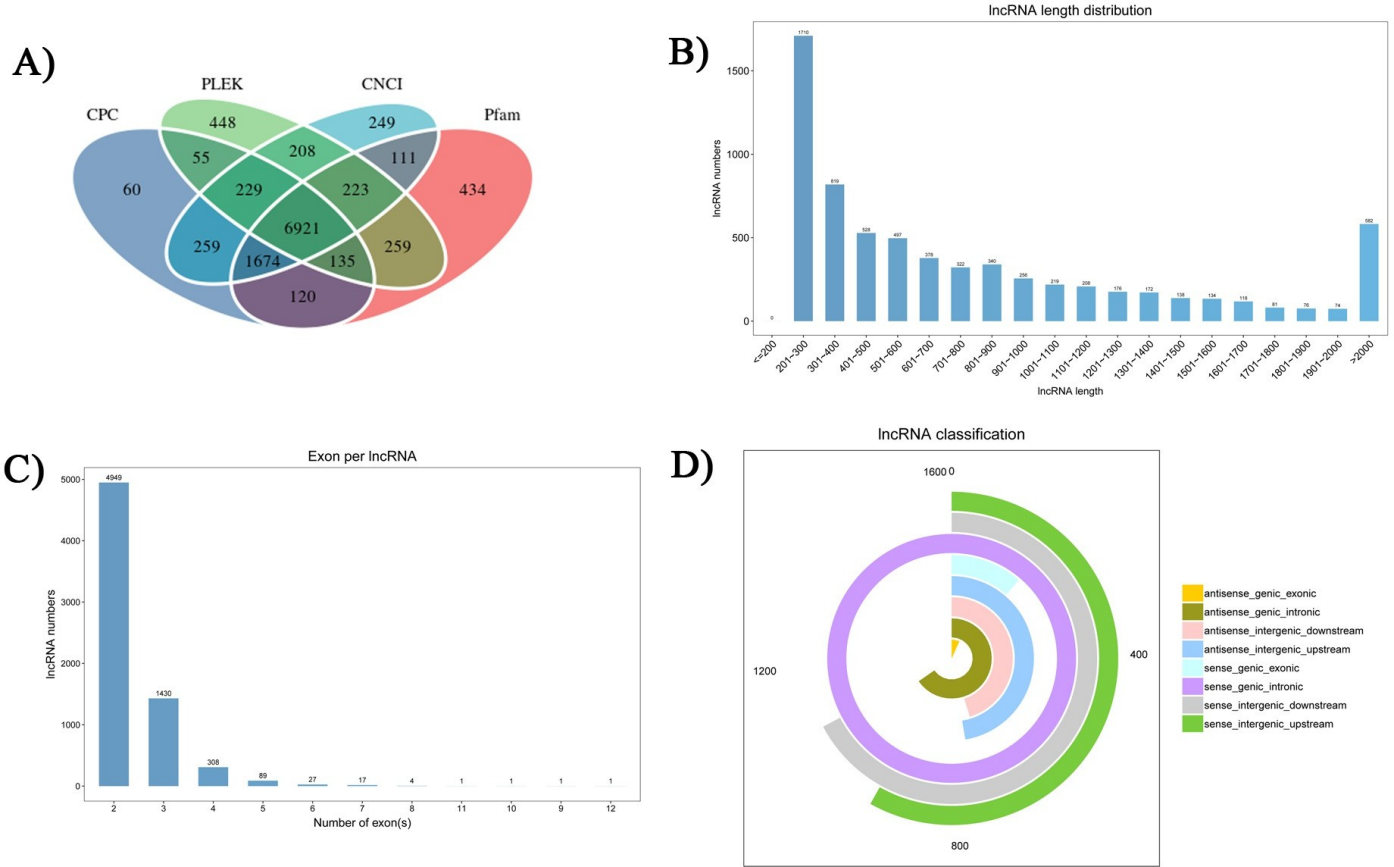
Features of *S*. *peregrina* lncRNAs. Venn diagram of novel long non-coding RNAs (lncRNAs) by CPC, CNCI, Pfam and PLEK analysis (A); the length distribution of lncRNAs (B); exon number distribution of lncRNAs (C); type distribution of lncRNAs (D).

The detailed identification results of lncRNAs in *S*. *peregrina* from CPC, CNCI, Pfam and PLEK are shown in S3 Table. By coding capacity analysis, these transcripts were predicted to have 6921 novel lncRNA transcripts (lncRNAs) (Fig 1A), with a total length of 5,836,143 nt and an average length of 854.74nt. Among them, the number of lncRNAs was the largest between 201 and 300nt (Fig 1B). Analysis of exon number distribution found that the most lncRNAs is exon 2 (4949). The second was lncRNAs with exon 3 (1430) (Fig 1C). The groups with the most lncRNAs were sense_genic_intronic (1623) and sense_intergenic_downstream (1091) (Fig 1D).

These lncRNAs were widely distributed on chromosomes, mostly on the chromosomes 1, 4, and 2 (S2 Fig). In addition, the expression levels of lncRNAs in different pupae tissue were calculated as FPKM values (S3A Fig). Since the correlation of transcript expression levels is an important indicator of the reliability of the experimental results, we found that the correlation coefficients between the three biological replicates of the three groups in this study, based on the expression of lncRNAs at different pupal stages, exhibited higher repeatability (S3B Fig).

### Differential expression of lncRNAs and mRNAs in *S*. *peregrina*

**Fig 2:**
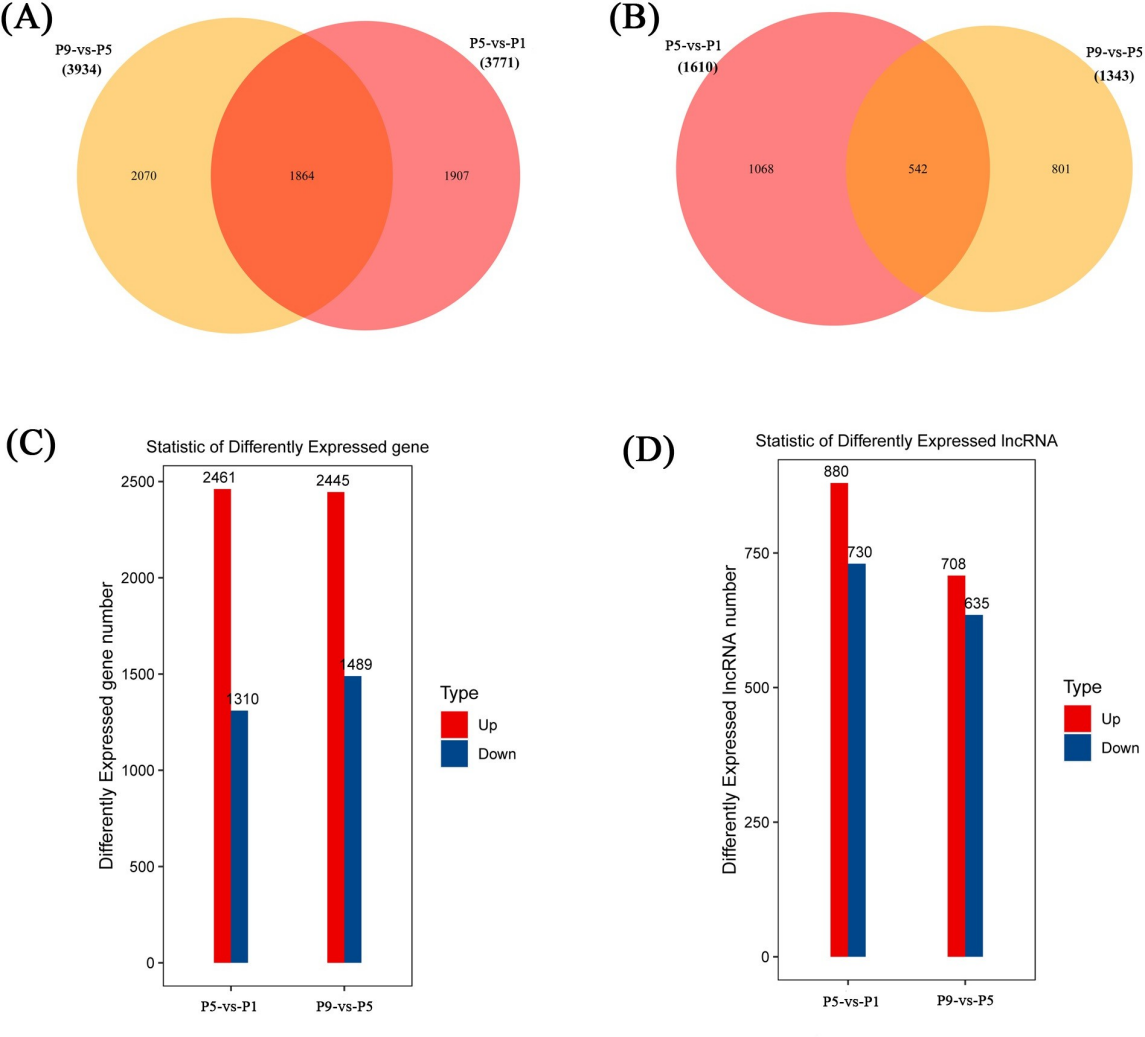
Expression Profiles of DE mRNAs and DE lncRNAs in three pupal developmental stages of *S*. *peregrina*. the Venn diagram showed the shared and unique genes of DE mRNAs (A) and DE lncRNAs (B); the Statistical histogram showed the DE mRNAs (C) and DE lncRNAs (D) number.

The differentially expressed mRNAs and lncRNAs were identified following the criteria of |log2(fold-change)| >1 and q < 0.05 as stated above. Intersection analysis showed that 1864 DE mRNAs (Fig 2A) and 542 DE lncRNAs (Fig 2B) overlapped between the three groups. There were 3771 DE mRNAs (2461 were upregulated, 1310 were downregulated) and 1610 DE lncRNAs (880 were upregulated, 730 were downregulated) were obtained between P5 and P1 groups, 3934 DE mRNAs (2445 were upregulated, 1489 were downregulated) and 1343 DE lncRNAs (708 were upregulated, 635 were downregulated) between P9 and P5 groups (Fig 2C and 2D). The detailed data of differentially expressed mRNAs and lncRNAs between P5-vs-P1 and P9-vs-P5 groups are shown in S4 Table.

Based on the expression among the three pupal developmental stages, DE lncRNAs were divided into eight statistically significant clusters, and time-series expression profiles of *S*. *peregrina* lncRNAs are shown in S3 Fig, including 169 DE lncRNAs in cluster two, 236 DE lncRNAs in cluster seven, 232 DE lncRNAs in cluster ten, 276 DE lncRNAs in cluster eleven, and 247 DE lncRNAs in cluster fourteen (S4A Fig). Cluster three showed the expression trend of 152 consecutively down-regulated genes (S4B and S4C Fig).

### Functional analysis of the differentially expressed lncRNAs and mRNAs

**Fig 3:**
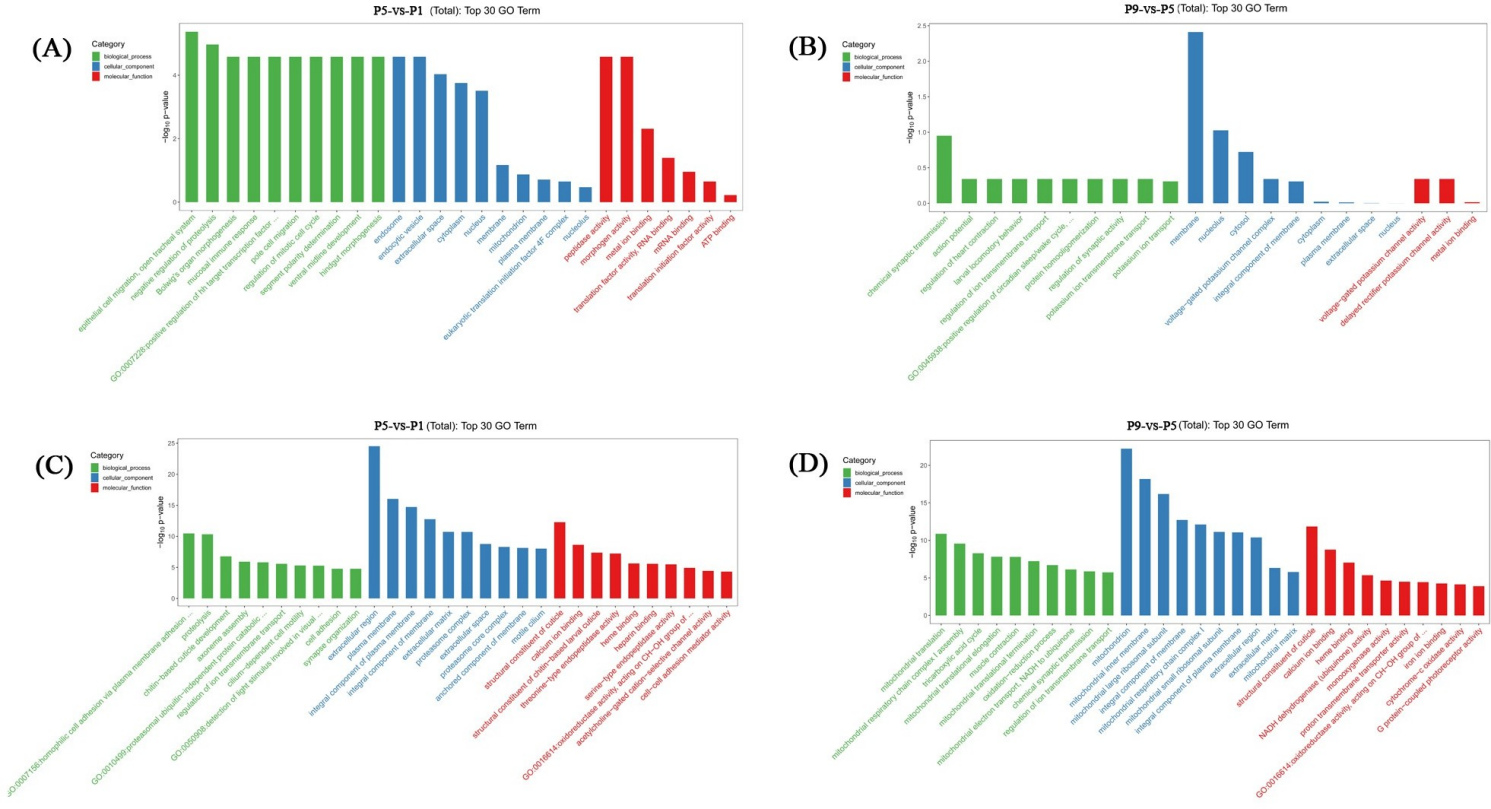
GO enrichment analysis of DE lncRNAs (A, B) and DE mRNAs (C, D), in the P5-vs-P1 comparison group and P9-vs-P5 comparison group.

To explore the putative functions of the DE lncRNAs and DE mRNAs, GO term and KEGG pathway analyses were performed. GO enrichment analysis showed that, DE lncRNAs in the P5-vs-P1 groups were mostly enriched in epithelial cell migration, open tracheal system, endosome, peptidase activity; DE lncRNAs in the P9-vs-P5 groups were enriched in chemical synaptic transmission, membrane, voltage-gated potassium channel activity (S5 Table and Fig 3A and 3B). In addition, GO enrichment analysis showed that, DE mRNAs in the P5-vs-P1 groups were mostly enriched in homophilic cell adhesion via plasma membrane adhesion molecules, extracellular region, structural constituent of cuticle, and DE mRNAs in the P9-vs-P5 groups were mostly enriched in mitochondrial translation, mitochondrion, structural constituent of cuticle (S6 Table and Fig 3C and 3D).

**Fig 4:**
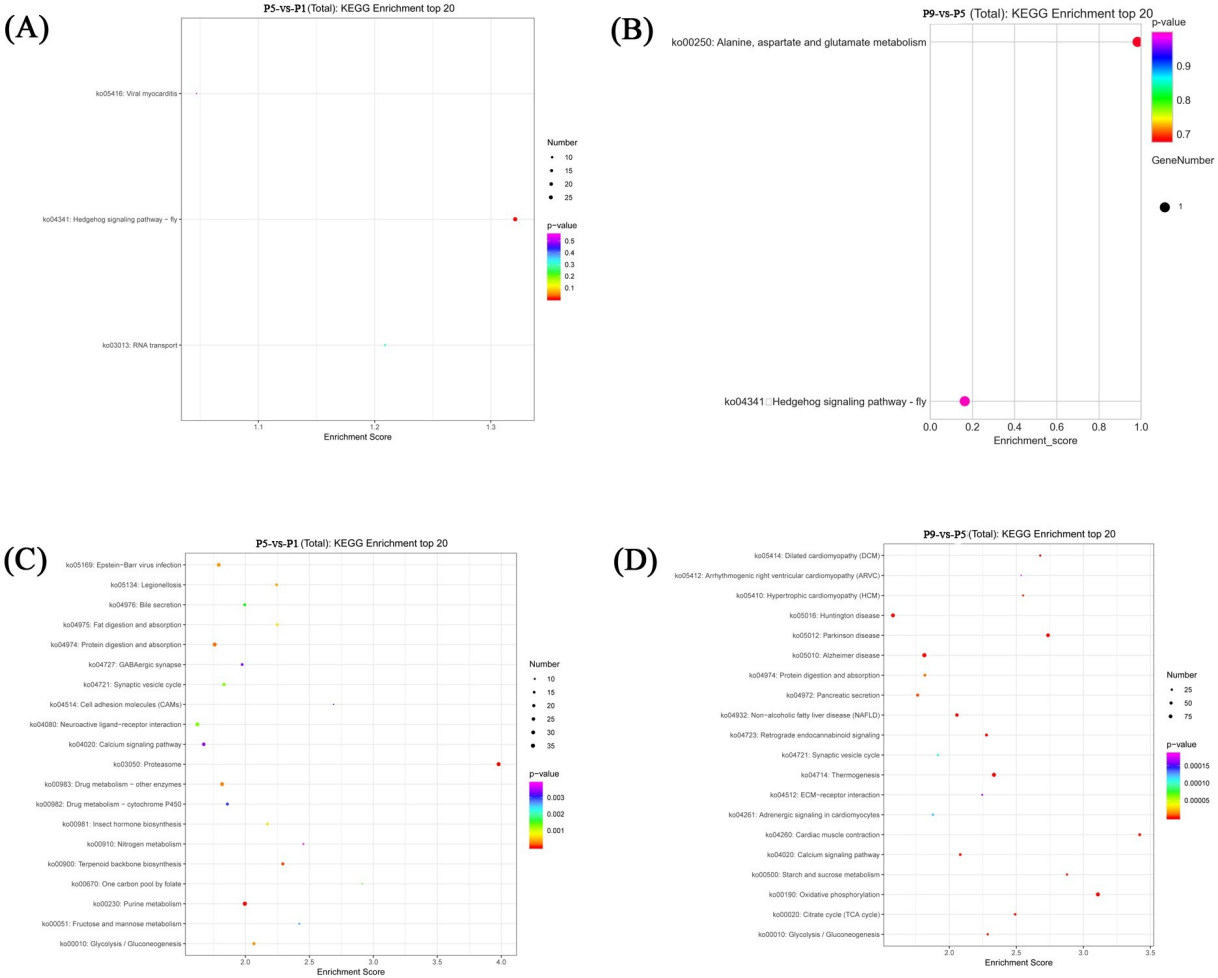
KEGG pathway analysis of DE lncRNAs (A, B) and DE mRNAs (C, D), in the P5-vs-P1 comparison group and P9-vs-P5 comparison group.

The results of KEGG pathway analysis showed that DE lncRNAs were mostly enriched in terms associated with Hedgehog signaling pathway—fly, RNA transport, viral myocarditis in the P5-vs-P1 groups, and Alanine, aspartate and glutamate metabolism, Hedgehog signaling pathway—fly in the P9-vs-P5 groups (S7 Table and Fig 4A and 4B). In addition, KEGG analysis showed that DE mRNAs were mostly enriched in Proteasome, Purine metabolism, Terpenoid backbone biosynthesis in the P5-vs-P1 groups, and Oxidative phosphorylation, Parkinson disease, Thermogenesis in the P9-vs-P5 groups (S8 Table and Fig 4C and 4D).

Specifically, functional enrichment analysis in the P5-vs-P1 groups showed that 25 of the same up-regulated DE lncRNAs were enriched in several GO terms associated with insect metamorphosis, such as ventral midline development, hindgut morphogenesis, and progression of morphogenetic furrow involved in compound eye morphogenesis, and were enriched in the KEGG pathway associated with Hedgehog signaling pathway—fly, of which, the DE lncRNA (*SP_lnc5000*) were up-regulated in the P5-vs-P1 groups, and were down-regulated in the P9-vs-P5 groups, and were enriched in the KEGG pathway of Hedgehog signaling pathway—fly, which might participate in regulating the pupal metamorphosis development of *S*. *peregrina*. The detailed information of Hedgehog signaling pathway—fly is shown in S5 Fig. GO enrichment analysis showed that 38 up-regulated DE mRNAs in the P5-vs-P1 groups and 51 up-regulated DE mRNAs in the P9-vs-P5 groups were enriched in structural constituent of cuticle.

### Co-expression network construction of DEGs

**Fig 5:**
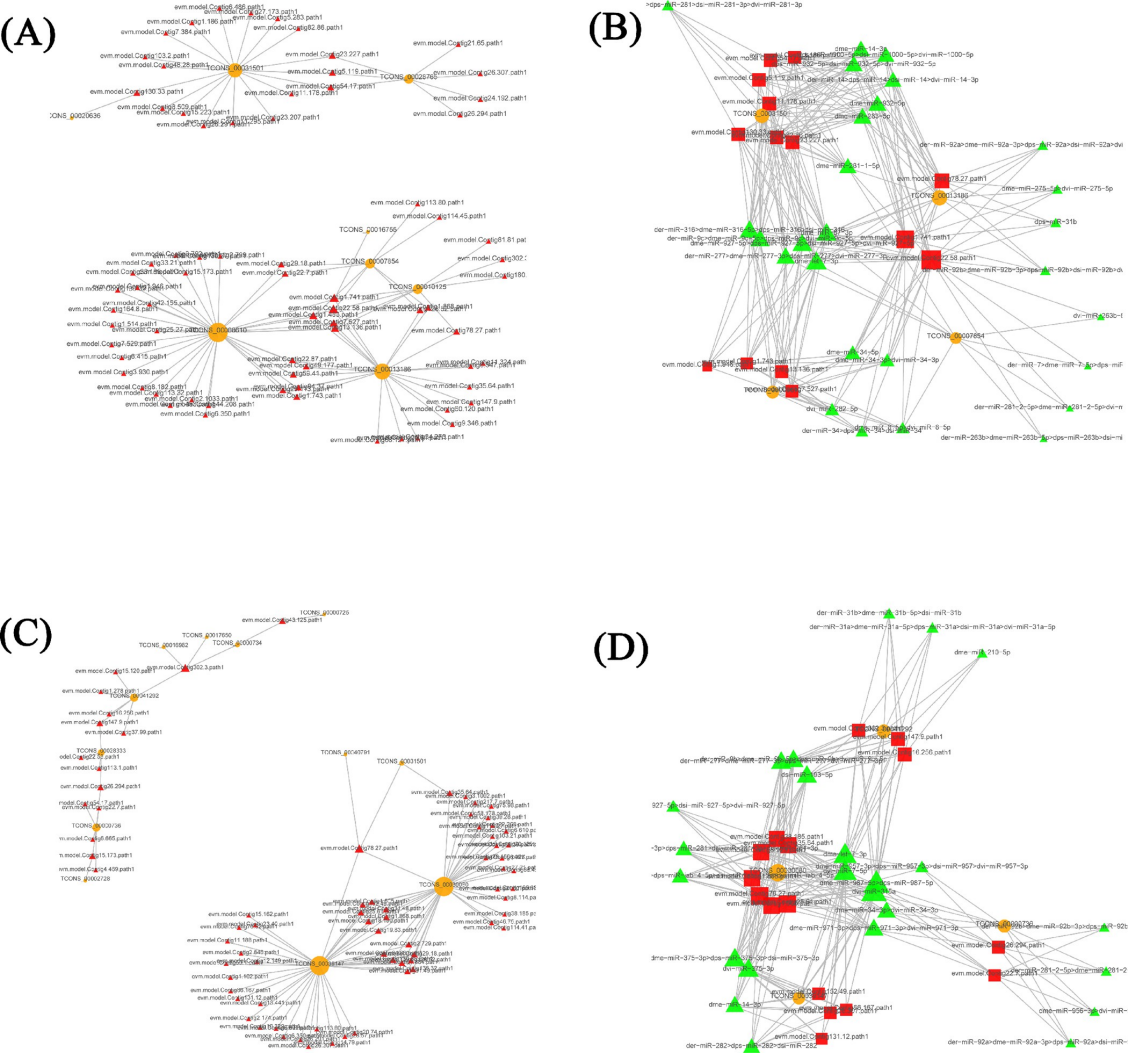
According to MuTATE score, we constructed the ceRNA regulatory networks of top100 lncRNA-mRNA pairs in the P5-vs-P1(A) and P9-vs-P5 (C) groups, and 200 lncRNA-miRNA-mRNA relationship pairs in the P5-vs-P1(B) and P9-vs-P5 (D) groups. In the lncRNA-mRNA networks: Orange circles indicate lncRNAs, Red triangles indicate mRNAs. In the lncRNA-miRNA-mRNA networks: Orange circles indicate lncRNAs, Red rectangles indicate mRNAs, and Green triangles indicate miRNAs.

In the present study, we chose 1610 DE lncRNAs, 62 DE miRNAs and 3771 DE mRNAs for co-expression analysis in the P5-vs-P1 groups, and 1343 DE lncRNAs, 41 DE miRNAs and 3934 DE mRNAs for co-expression analysis in the P9-vs-P5 groups. S9 Table shows all lncRNA-mRNA and lncRNA-miRNA-mRNA relationship pairs in the P5-vs-P1 groups and P9-vs-P5 groups. According to MuTATE score, ceRNA analysis of the top100 lncRNA-mRNA pairs (Fig 5A and 5C), and the 200 lncRNA-miRNA-mRNA relationship pairs (Fig 5B and 5D) are shown, and the results suggests that a total of 100 lncRNA-mRNA axes were built up including 73 mRNAs and 8 lncRNAs, and 235 lncRNA-miRNA-mRNA axes were built up including 16 mRNAs, 27 miRNAs, and 4 lncRNAs, in the P5-vs-P1 comparison groups. Specifically, the DE lncRNA (*SP_lnc5000*) may participate in regulating the pupal metamorphosis of *S*. *peregrina* by a lncRNA-miRNA-mRNA co-expression network.

### Validation of differentially expressed lncRNAs and mRNAs by RT-qPCR

**Fig 6:**
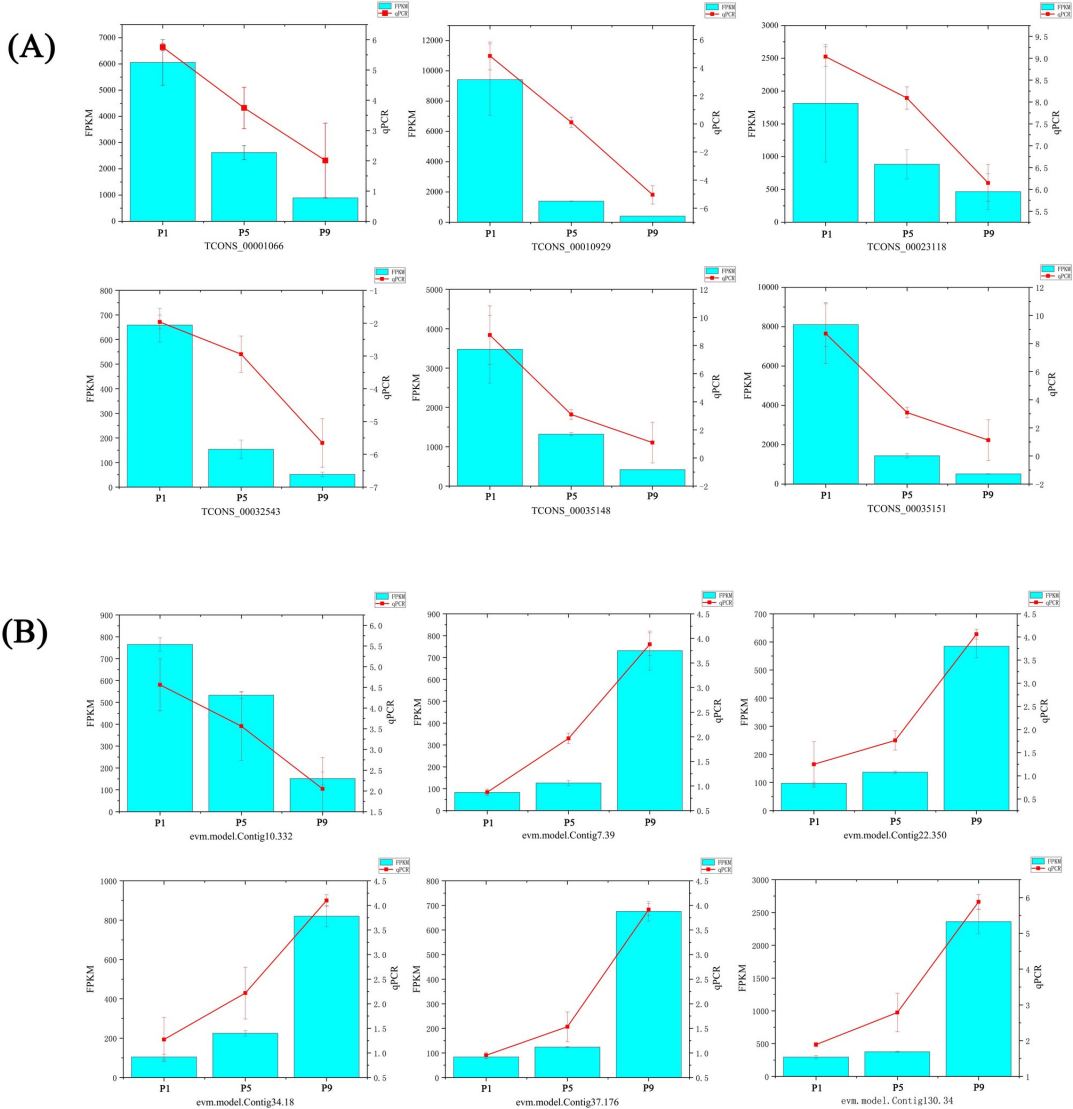
RNA-seq data validation of DE lncRNAs (A) and DE mRNAs (B) by quantitative real-time PCR (RT-qPCR). The data are presented as the mean ± SD. The right left Y-axis indicates the relative expression levels calculated by qPCR and the left Y-axis indicates the FPKM values of RNA-seq data.

To validate the RNA-seq results, six DE mRNAs (evm.model.Contig10.332, evm.model.Contig130.34, evm.model.Contig22.350, evm.model.Contig34.18, evm.model.Contig7.39, evm.model.Contig37.176) and six DE lncRNAs (TCONS_00001066, TCONS_00010929, TCONS_00023118, TCONS_00032543, TCONS_00035148, TCONS_00035151) from the different developmental times of *S*. *peregrina* pupae were subjected to RT-qPCR. As shown in Fig 6, a comparative analysis of all the selected genes showed a consistent expression pattern in the RT-qPCR analysis as observed in RNA-seq data.

### Effects of *SP_lnc5000* RNAi on development of *S*. *peregrina*

**Fig 7:**
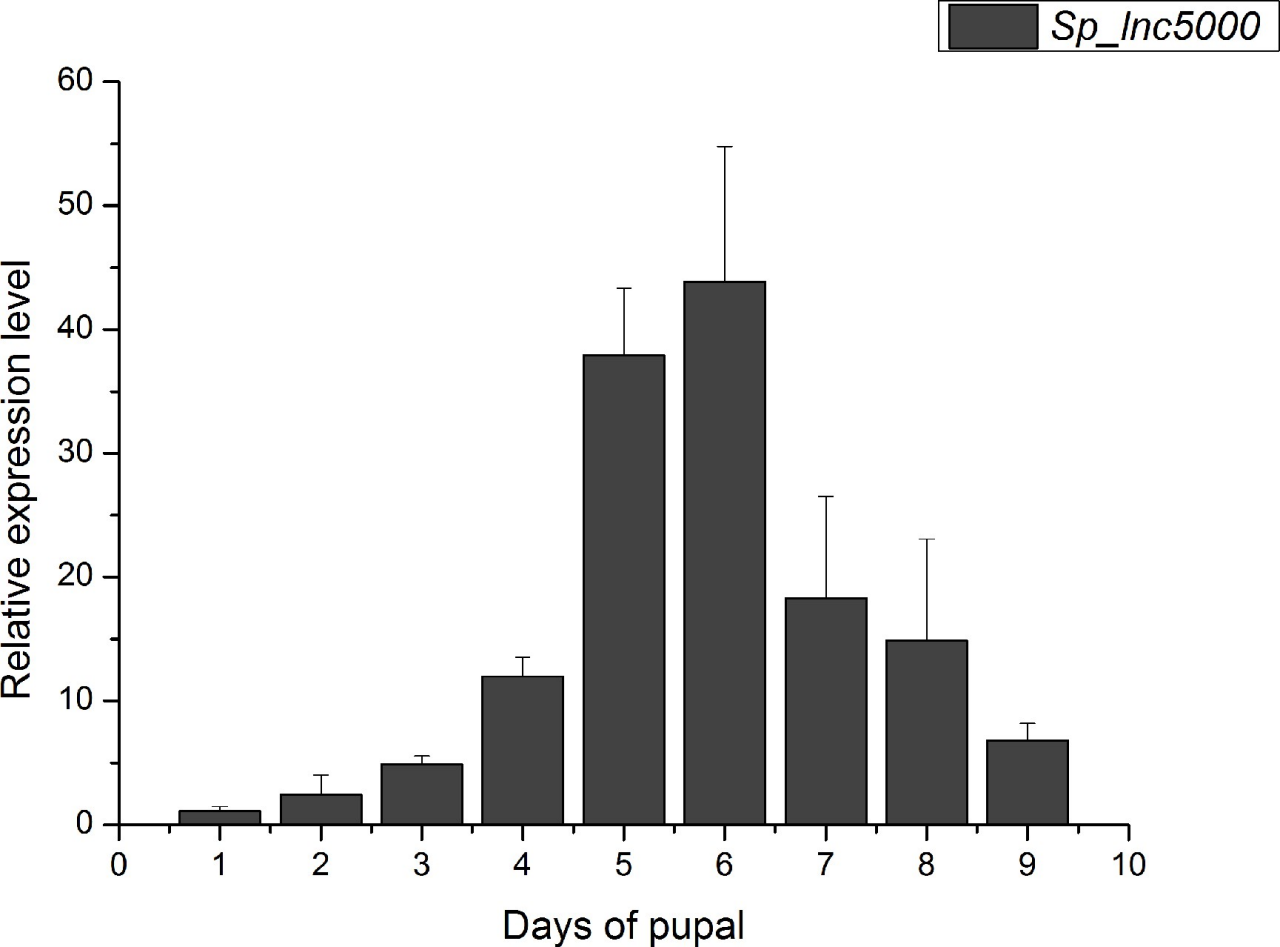
The developmental expression patterns of *Sp_lnc5000* during each day in the pupa stage (days 1–9) of *S*. *peregrina* by RT-qPCR. All data are showed as means ± SD.

**Fig 8:**
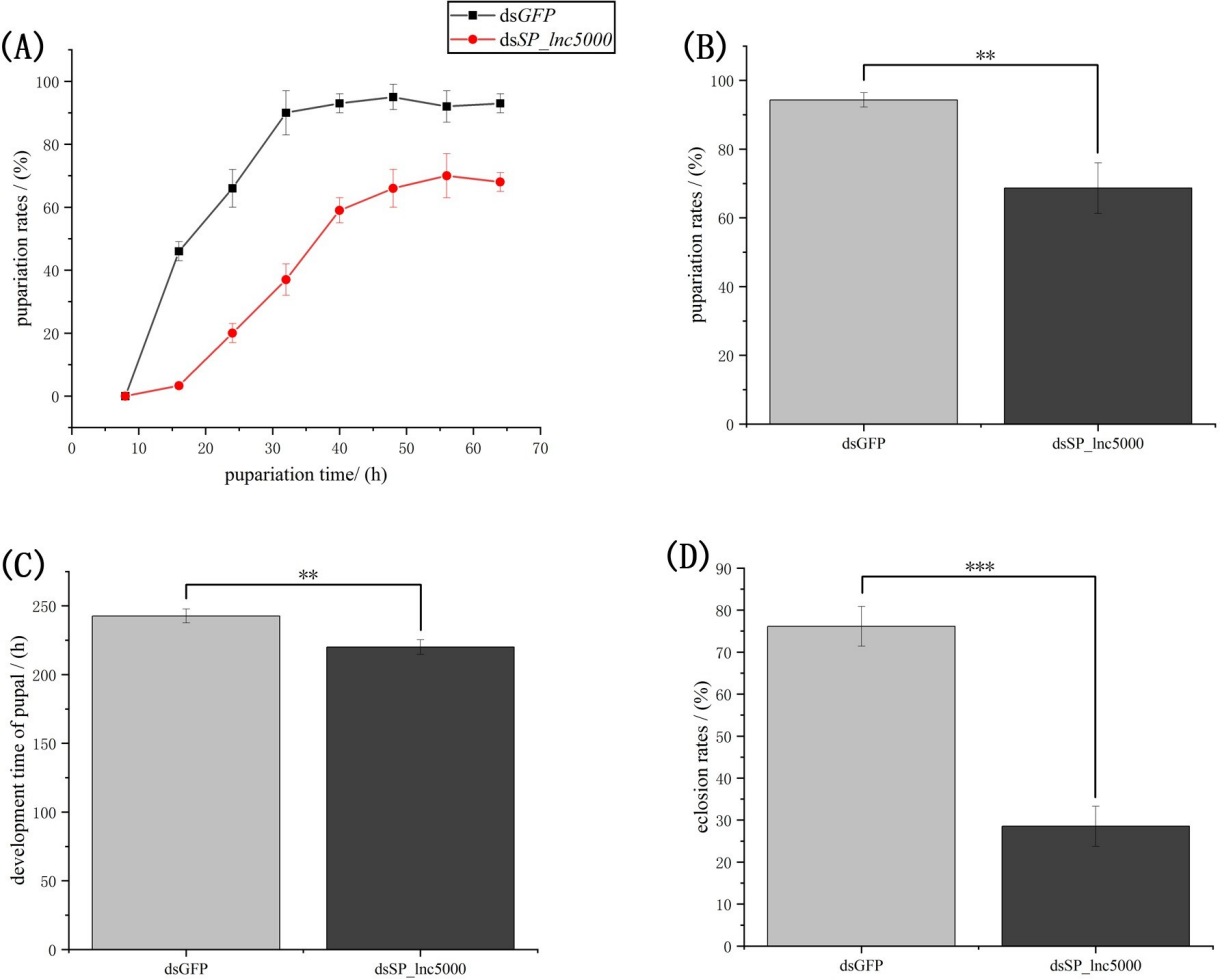
Effect of *Sp_lnc5000* double-stranded RNA (dsRNA) injection on development of S. peregrina, including the pupariation time (A), pupariation rates (B), the development time of pupal stage (C) and eclosion rates (D). dsGFP is used as the negative control; All data are reported as means ± SD, (*, P < 0.05; **, P < 0.01; ***, P < 0.001).

**Fig 9:**
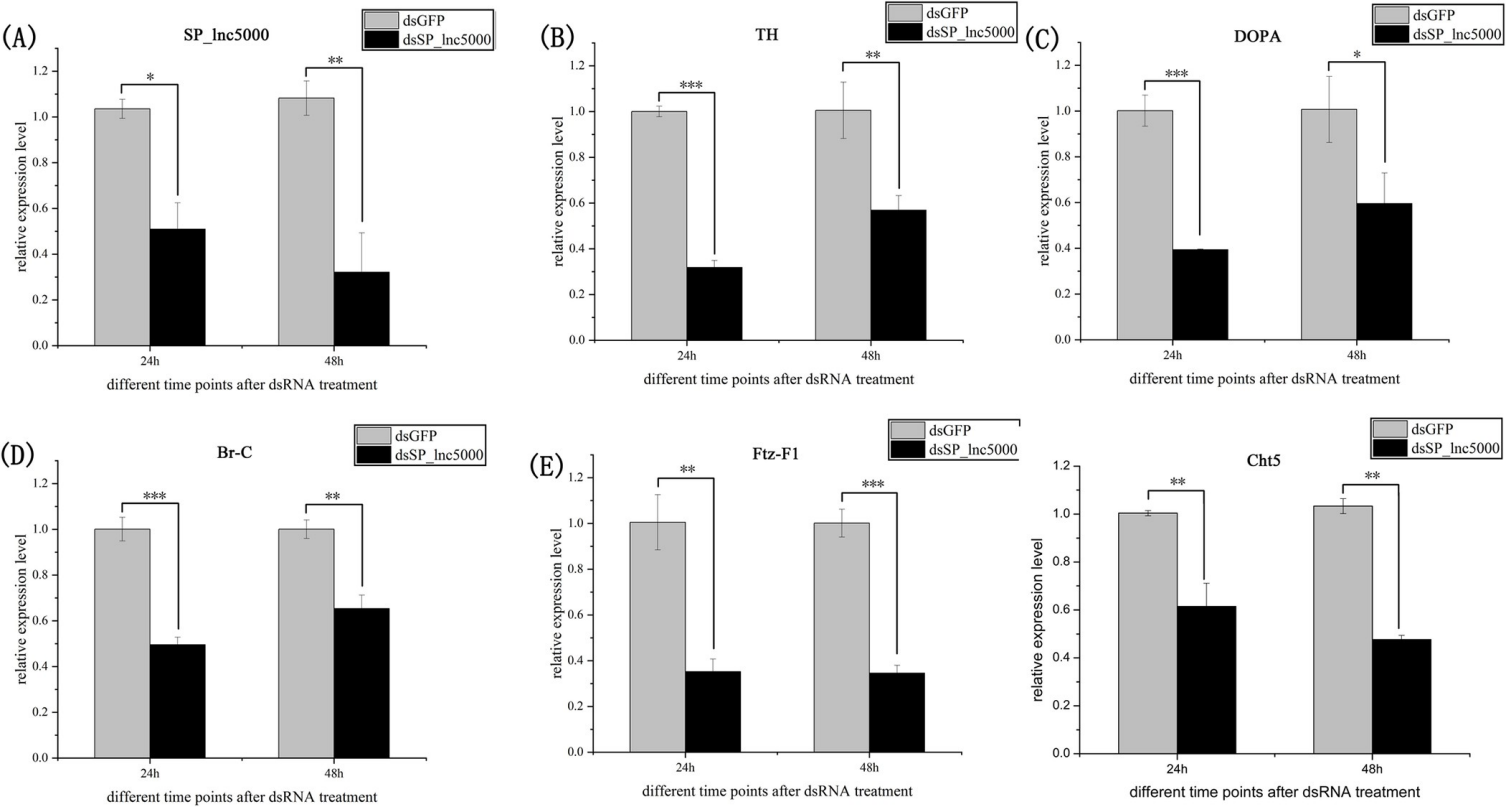
RNAi efficiency after injected ds*Sp_lnc5000* for 24h and 48 h (A). The relative expression level change of four TH, DOPA, Br-c, Ftz-F1, Cht5 genes after injected ds*Sp_lnc5000* on 24h and 48 h (B-F). dsGFP is used as the negative control for RNAi; actin is used as an internal reference for qPCR. All data are reported as means ± SD, (*, P < 0.05; **, P < 0.01; ***, P < 0.001).

To identify the lncRNAs that may regulate development of *S*. *peregrina*, we selected a DE lncRNA (*SP_lnc5000*) from the RNA-seq results. The results of developmental expression patterns of *SP_lnc5000* during each day in the pupa stage (days 1–9) shown that expression peaked at mid-term pupal stage and then decreased to a relatively low level before eclosion (Fig 7). After injection with ds*SP_lnc5000*, compared with the dsGFP control group, the pupariation rate was decreased to 68% compared with 94% of the control group, the eclosion rate was decreased to 28% compared with 76% of the control group, and the development time of pupal stage was decreased to 220h compared with 242h of the control group (Fig 8). The relative expression level of *SP_lnc5000* was significantly decreased to 50% and 32% at 24h and 48h post injection of *SP_lnc5000* as compared with the dsGFP control group (Fig 9). Similarly, several insect metamorphosis development related genes, including 20-hydroxyecdysone (20E) signaling (Br-c, Ftz-F1), puparium tanning pathway (TH, DOPA), and chitin related pathway (Cht5), presented significantly declined trends after 24h and 48h of injections dsRNA of *SP_lnc5000* (Fig 9).

**Fig 10:**
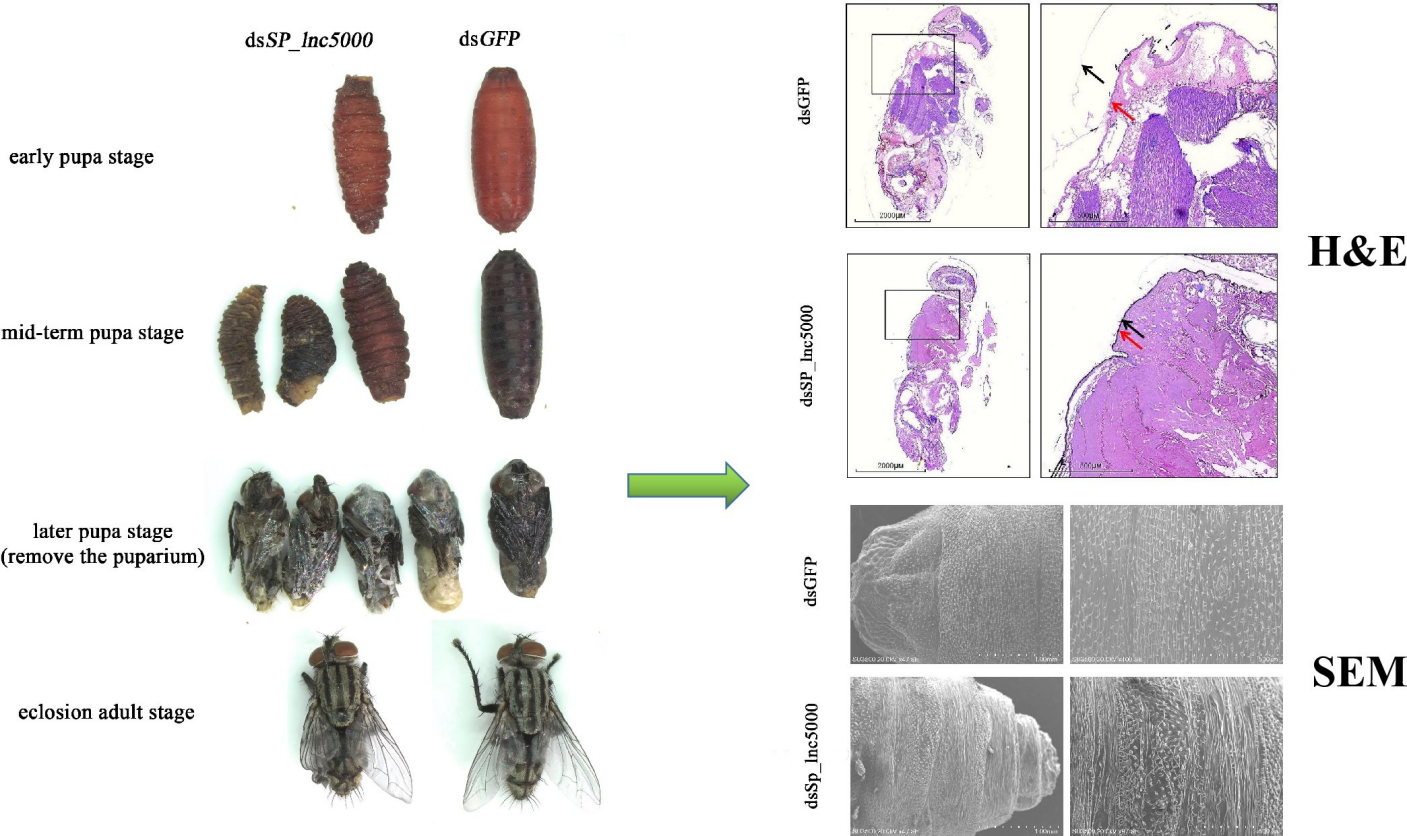
The morphological changes of *S*. *peregrina* after injected with dsSP_lnc5000, compared with dsGFP control group. The left side shows the phenotypic changes after injected with dsSP_lnc5000, including deformed pupae that cannot emerge, rough and asymmetrical puparium, deeply wrinkled pupal segment, stagnated or defect in pupal-adult metamorphosis, and wing-defective phenotypes of adult. H&E staining shows that the pupal cuticle and adult epidermal stuck closely together and the separation failed after injected with dsSP_lnc5000, the black arrow represents the pupa cuticle, the red arrow represents the adult epidermal. Scanning electron microscopy (SEM) shows that the intersegmental spines of pupal cuticle crowded, mixed, disordered, and varies in size after injected with dsSP_lnc5000.

The phenotypic changes of *S*. *peregrina* between the ds*SP_lnc5000* group and the dsGFP control group at different development stages were observed under a stereomicroscope. Injection of dsRNA of *SP_lnc5000* during the late larvae stage resulted in deformed pupae that could not emerge, rough and asymmetrical puparium, deeply wrinkled pupal segment, stagnated or defect in pupal-adult metamorphosis, and wing-defective phenotypes of adult (Fig 10). At later pupal stage after ds*SP_lnc5000*-injection, we observed that the inner surface of the puparium was covered with a large transparent membrane (Fig 10). Furthermore, H&E staining results showed that the pupal cuticle and adult epidermal could be clearly separated in the control group, while they were stuck closely together and the separation failed in the interference groups (Fig 10). The results of SEM showed that the intersegmental spines on the pupal cuticle of the control group were triangular and arranged evenly, neatly, and orderly, while in the experimental groups they were crowded, mixed, and disordered, and the intersegmental spines were different in size (Fig 10). This observation indicated that *SP_lnc5000* might influence metamorphosis development of *S*. *peregrina* by interfering with the structural components of the pupal cuticle.

**Fig 11:**
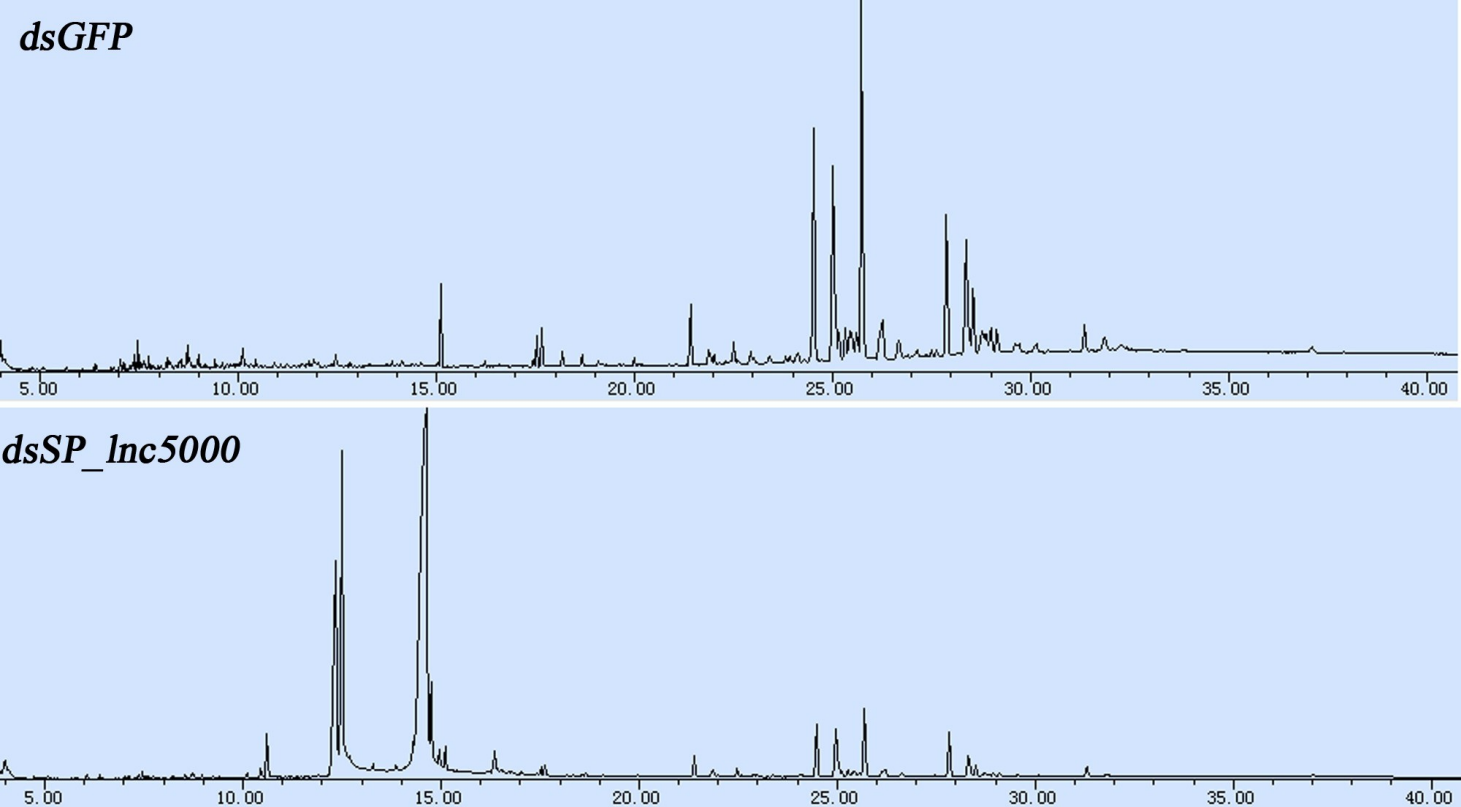
GC chromatograms showing CHC profiles of the pupal cuticle at later pupa stage after injected with dsGFP and ds*Sp_lnc5000*.

Fig 11 shows the GC chromatograms of *S*. *peregrina* of the pupal cuticle at later pupal stage after injection with dsGFP and ds*SP_lnc5000*. The CHCs detected in the chromatograms are given in Table 2. After injected ds*SP_lnc5000*, a total of 33 CHCs were identified by GC–MS analysis in the CHC profiles of the *S*. *peregrina* pupal, including 7 n-alkanes, 24 branched alkanes and 2 alkenes compounds with a carbon chain length between C17 and C35, while 37 CHCs were identified in the dsGFP control group, including 10 n-alkanes, 25 branched alkanes and 2 alkenes compounds with a carbon chain length between C14 and C35. The n-alkanes and branched alkanes were the two primary compounds of all detected hydrocarbons. We found that the number of n-alkanes was decreased, the number and concentration of CHCs with a longer carbon chain were much reduced post injection of ds*SP_lnc5000* as compared with the dsGFP control group. The C21 and 6-Methyl C19 alkane were the most dominant n-alkanes and branched alkanes in the ds*SP_lnc5000* group, compared with C29 and 3-Methyl C29 being the most dominant compounds in the dsGFP group. The results indicated that *SP_lnc5000* appears to influence metamorphosis developmental of *S*. *peregrina* through affecting the composition of compounds of the pupal cuticle.

**Table 2:** CHC profile of *S*. *peregrina* puparium after injected with dsGFP and ds*SP_lnc5000*.

			% composition (mean±SD)	
Peak number	Peak identification	Kovats Index	dsGFP	ds*SP_lnc5000*
1	C14	1400	0.62±0.02	-
2	C17	1700	1.16±0.07	1.41±0.27
3	6-Methyl C17	1750	-	0.6±0.04
4	C19:1	1893	-	0.59±0.06
5	C19	1900	-	0.6±0.02
6	6-Methyl C19	1951	-	32.19±6.04
7	C21:1	2098	-	2.7±0.56
8	C21	2100	-	1.82±0.5
9	DiMethyl-C21	2207	2.8±0.98	-
10	C25	2500	0.76±0.12	-
11	Methyl C25	2531	-	0.74±0.03
12	11-Methyl C25	2536	-	0.61±0.07
13	C26	2600	-	0.63±0.11
14	C27	2700	2.87±0.28	-
15	Methyl C27	2730	1.02±0.02	1.74±0.24
16	9-Methyl C27	2737	0.71±0.03	0.79±0.05
17	11,15-DiMethyl C27	2762	0.59±0.17	-
18	3-Methyl C27	2771	1.22±0.12	-
19	C28	2800	0.86±0.1	0.73±0.12
20	4-Methyl C28	2853	0.66±0.02	0.61±0.09
21	3-Methyl C28	2875	0.76±0.08	0.7±0.1
22	C29	2900	11.51±0.7	-
23	Methyl C29	2929	9.57±0.33	8.43±0.04
24	13-Methyl C29	2931	-	2.85±0.49
25	7-Methyl-C29	2942	-	1.88±0.39
26	13,17-DiMethyl C29	2952	1.62±0.18	-
27	5-Methyl C29	2955	1.67±0.12	1.44±0.35
28	9,15-DiMethyl C29	2961	-	1.17±0.11
29	11,15-DiMethyl C29	2964	1.73±0.08	-
30	DiMethyl C29	2971	-	15.73±3.72
31	3-Methyl C29	2972	18.01±0.48	-
32	C30	3000	-	1.73±0.13
33	14-Methyl C30	3030	1.49±0.09	-
34	8-Methyl C30	3035	0.71±0.03	-
35	6-Methyl C30	3039	0.76±0.03	-
36	8,14-DiMethyl C30	3051	-	0.56±0.05
37	2-Methyl C30	3065	0.72±0.03	-
38	C31:1	3081	0.93±0.05	-
39	2,10-DiMeC31	3094	-	7.23±1.24
40	C31	3100	7.36±0.53	-
41	Cholesterol	3124	-	3.62±0.77
42	2,6,14-Trimethyl C30	3125	7.63±1.15	-
43	9-Methyl C31	3134	-	3.39±1.1
44	7-Methyl C31	3141	3.98±0.2	-
45	7,15-DiMethyl C31	3148	-	0.94±0.05
46	8,14-DiMethyl C31	3152	1.89±0.06	-
47	13,17-DiMethyl-C31	3154	1.77±0.11	0.73±0.1
48	11,21-DiMethyl C31	3161	-	1.12±0.19
49	7,11-DiMethyl C31	3164	1.98±0.21	-
50	5,13-DiMethyl C31	3170	-	1.03±0.08
51	C32:1	3173	2.15±0.2	-
52	C32	3200	1.3±0.09	0.53±0.06
53	11-Methyl C32	3229	1.34±0.16	0.6±0.01
54	C33	3300	2.08±0.08	-
55	5-Methyl C33	3350	1.43±0.18	-
56	11,15-DiMethyl C33	3359	1.71±0.64	-
57	C34	3400	1.24±0.23	-
58	11,23-DiMethyl C35	3557	1.37±0.17	0.57±0.12

-: Not detected or compounds with peak area percentage below 0.5%

## Discussion

*Sarcophaga peregrina*, a significant flesh fly species, in hygiene pest management, as vectors of parasitic disease agents, can cause myiasis and other tropical infectious diseases [20], and have been implicated in the transmission of diseases such as cutaneous leishmaniasis. Understanding the metamorphosis of *S*. *peregrina* could lead to the identification of potential targets for vector control and the development of effective strategies for controlling the spread of parasitic diseases.

The metamorphosis of insects involves many genes and signaling pathways [50]. To understand the regulatory mechanisms of this process, it is necessary to analyze expression patterns of numerous genes [51]. Recent studies have shown that lncRNAs play a role in almost all aspects of insect development, reproduction, and genetic plasticity [52]. There is a need to expand lncRNA studies in wider variety of insects in order to acquire a better understanding of the connections between lncRNA and the metamorphosis of insects. In the present study, to better understand the regulatory mechanism of lncRNAs in *S*. *peregrina* metamorphosis, we first performed genome-wide identification and characterization of lncRNAs from three different pupal developmental stages of *S*. *peregrina* by RNA-seq, and identified approximately 6921 novel lncRNAs transcripts from these samples. This number is similar to the lepidopteran, *Bombyx mori* [9], and greater than for some other insect species, including *Aedes aegypti* [10], *Plutella xylostella* [11] and *Apis mellifera* [12]. This difference may be caused by genome size, different samples, and different annotation strategies [53]. The lncRNAs found in our investigation on *S*. *peregrina* shared known characteristics of lncRNAs from other species, including shorter size, lower GC content and low sequence conservation, according to genomic features.

Insect studies have found that many differentially expressed genes, including lncRNA, are involved in regulating metamorphosis development [54]. In our study, enrichment analysis of *S*. *peregrina* showed that DE lncRNAs were enriched in several associated with insect metamorphosis, such as ventral midline development, and hedgehog signaling pathway—fly. Studies have shown that the hedgehog signaling pathway is essential for metazoan development and tissue homeostasis [55]. The results of gene expression analysis suggest that DE lncRNAs might be associated with insect metamorphosis development. In addition, the temporal specificity of lncRNA expression suggests that they may perform unique biological tasks at specific developmental stages [56] We found a DE lncRNA (*SP_lnc5000*) was up-regulated in the P5-vs-P1 groups, and down-regulated in the P9-vs-P5 groups, and expression peaked at mid-term pupal stage, which suggests that *SP_lnc5000* may play an important role in the mid-term pupal stage.

In this study, we presented the role of lncRNAs in the metamorphosis of *S*. *peregrina*, and identified a molecular target that could potentially be used in pest control. We found a DE lncRNA (*SP_lnc5000*) might participate in regulating the metamorphosis of *S*. *peregrina*. When knocking down expression of the *SP_lnc5000* genes by RNAi, we found a significant effect on metamorphosis development of the pupal cuticle, resulting in deformed pupae that could not emerge, rough and asymmetrical puparium, deeply wrinkled pupal segment, stagnated or defect in pupal-adult metamorphosis, and wing-defective phenotypes of adult, and observed that the surface of the intra-puparial body was covered with a large transparent membrane (Fig 10). H&E staining results showed that the pupal cuticle and adult epidermis stuck closely together, and failure to degrade the pupal cuticle, might result in its emergence failure. SEM observation confirmed the abnormal development in the intersegmental spines structure on the pupal cuticle from the microscopic perspective. The cuticle is the outermost part of the insect body, and is a key determinant in maintaining insect locomotion, mechanical support, body shape and normal development [57]. The cuticle periodically degrading and rebuilding is important for growth during molting and metamorphosis of insects [58]. During insect metamorphosis, cuticle degradation undergoes two distinct but causally related processes. First, the larval cuticle degrades to form the pupal cuticle, and second, the pupal cuticle degrades to form the adult epidermis [15]. Abnormal structure development or failure of cuticle degradation can seriously affect metamorphosis in insects.

In addition, cuticular hydrocarbons (CHCs) are the main components of the insect cuticle, and they plays important roles in the formation of the insect cuticle, and in the growth development of insects [59]. Several studies have shown that the arrangement of CHCs crystals from longer chains, which have higher melting temperatures, provides better barriers against water loss, and increasing chain length strengthens van der Waals forces and leads to stability [60]. In our study, GC-MS analysis found that the composition and concentration of CHCs of the pupal cuticle significant changed post injection of ds*SP_lnc5000*, the number and concentration of CHCs with a longer carbon chain were much reduced, which may affect the stability of the pupal cuticle and further affect the development of insect.

The insect molting hormone 20-hydroxyecdysone (20E) promotes metamorphosis by upregulating 20E-pathway gene expression and by counteraction with the juvenile hormone and insulin [61], and the steroid hormone 20E also promotes programmed cell death (PCD) during pupal metamorphosis in insects [62]. In our study, RNA interference of *SP_lnc5000* presented significantly declined expression of 20E signaling pathway related genes, which may lead to failure or obstruction of apoptosis processes, then pupal cuticle and adult epidermis stuck closely together, and impeded the pupal cuticle degradation process, resulting in its metamorphosis failure. This is consistent with our experimental observations, and sheds light on the importance of 20E in the regulation of insect metamorphosis, and its potential role in the control of insect pests.

Based on these studies, we speculated that *SP_lnc5000* might influence metamorphosis of *S*. *peregrina* through affecting the structure, apoptosis processes, and composition of compounds of the pupal cuticle, and play an essential function in pupal metamorphosis. Importantly, our study also provides a reference for the potential utilization of key genes in the metamorphosis process, which is indispensable for *S*. *peregrina* subsistence in developing effective control strategies.

However, research has found that some potential confounding factors, such as fluctuating temperature, humidity, light, and microorganisms, can also affect the metamorphosis of insects [63]. For example, changes in temperature and light can cause insect diapause [64]. Additionally, sexual dimorphism can also influence the growth and development of flies [64,65]. Although we used random sampling and mixing multiple samples to minimize the impact of sexual dimorphism on experimental results, it is still necessary to clarify the regulatory mechanisms of lncRNAs in male and female flies in the future, in order to better develop related insect management strategies. Differences in dsRNA degradation, cellular uptake, inter- and intracellular transports, processing of dsRNA to siRNA, and RNA-induced silencing complex formation influence RNAi efficiency [66]. Major challenges to widespread use of RNAi in insect pest management include variable RNAi efficiency among insects, lack of reliable dsRNA delivery methods, and off-target and nontarget effects, need to be further studied in the future.

## Supporting information

S1 Fig Morphological pictures of *S*. *peregrina* pupae in the early pupa stage (1 days pupae), mid-term pupa stage (5 days pupae), and later pupa stage (9 days pupae) at 25°C developmen. (PDF)

S2 Fig The chromosome distribution of lncRNAs of *S*. *peregrina*. (PDF)

S3 Fig The expression level distribution of lncRNAs identified (A), the correlation coefficient between three replicate samples based on lncRNA expression (B), in pupae tissues of *S*. *peregrina*.
(PDF)

S4 Fig Time-series expression profiles of DE lncRNAs of *S*. *peregrina* (A); Significant trend chart (B) and cluster heat map (C) of cluster three, the y-axis indicates the expression value after homogenization, and the x-axis indicates the developmental stage, including the early pupa stage (P1), mid-term pupa stage (P5), and later pupa stage (P9).
(PDF)

S5 Fig Insect Hedgehog signaling pathway—fly. (PDF)

S1 Table Sequencing quality statistics in *S*. *peregrina*. (XLSX)

S2 Table Sequencing reference genome comparisons in *S*. *peregrina*. (XLSX)

S3 Table The detailed identification results of lncRNAs in *S*. *peregrina* from CPC, CNCI, Pfam and PLEK. (XLSX)

S4 Table The differentially expressed mRNAs between P5-vs-P1 groups (A); The differentially expressed lncRNAs between P5-vs-P1 groups (B); The differentially expressed mRNAs P9-vs-P5 groups (C); The differentially expressed lncRNAs between P9-vs-P5 groups (D).
(XLSX)

S5 Table GO enrichment analyses of the total DE lncRNAs (A), up-regulated DE lncRNAs (B), down-regulated DE lncRNAs (C), in the P5-vs-P1 comparison group; GO enrichment analyses of the total DE lncRNAs (D), up-regulated DE lncRNAs (E), down-regulated DE lncRNAs (F), in the P9-vs-P5 comparison group.
(XLSX)

S6 Table GO enrichment analyses of the total DE mRNAs (A), up-regulated DE mRNAs (B), down-regulated DE mRNAs (C), in the P5-vs-P1 comparison group; GO enrichment analyses of the total DE mRNAs (D), up-regulated DE mRNAs (E), down-regulated DE mRNAs (F), in the P9-vs-P5 comparison group.
(XLSX)

S7 Table KEGG pathway analyses of the total DE lncRNAs (A), up-regulated DE lncRNAs (B), down-regulated DE lncRNAs (C), in the P5-vs-P1 comparison group; KEGG pathway analyses of the total DE lncRNAs (D), up-regulated DE lncRNAs (E), down-regulated DE lncRNAs (F), in the P9-vs-P5 comparison group.
(XLSX)

S8 Table KEGG pathway analyses of the total DE mRNAs (A), up-regulated DE mRNAs (B), down-regulated DE mRNAs (C), in the P5-vs-P1 comparison group; GO enrichment analyses of the total DE mRNAs (D), up-regulated DE mRNAs (E), down-regulated DE mRNAs (F), in the P9-vs-P5 comparison group.
(XLSX)

S9 Table All lncRNA-mRNA (A) and lncRNA-miRNA-mRNA (B) relationship pairs in the P5-vs-P1 groups; lncRNA-mRNA (C) and lncRNA-miRNA-mRNA (D) relationship pairs in the P9-vs-P5 groups.
(XLSX)
