# Supplementary figures and images for: Genome-wide analysis of long noncoding RNAs and their association in regulating the metamorphosis of the Sarcophaga peregrina (Diptera: Sarcophagidae)
Source: PLoS Negl Trop Dis. 2023 Jun 26;17(6):e0011411. doi: 10.1371/journal.pntd.0011411 (PMC10328366; doi:10.1371/journal.pntd.0011411)

**1 days pupa**

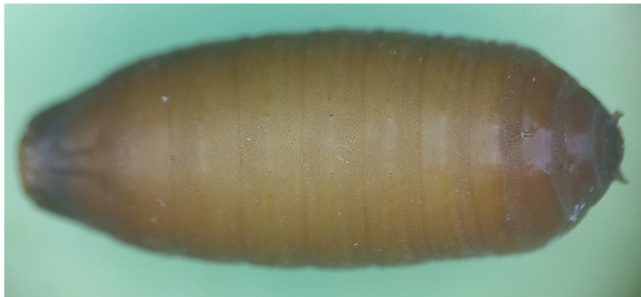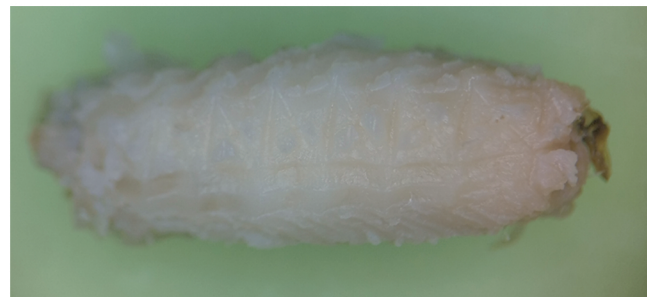

**5 days pupa**

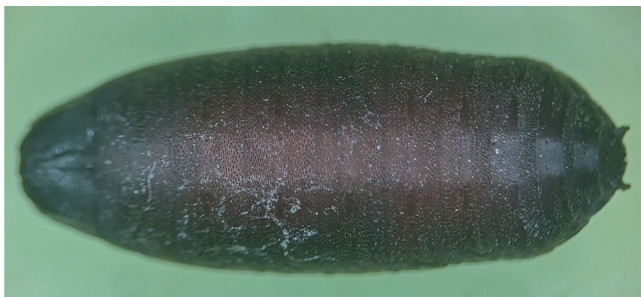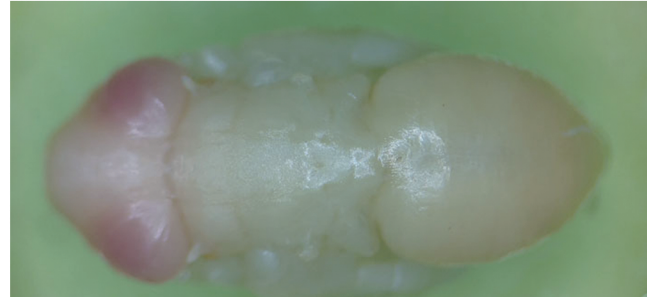

**9 days pupa**

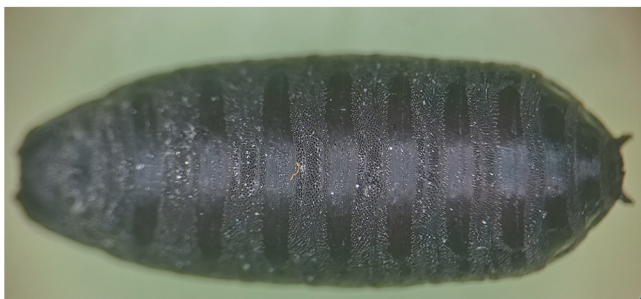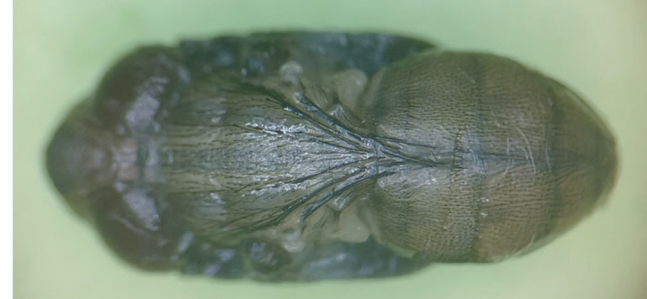

Supplement: S1 Fig — (PDF) [file pntd.0011411.s001.pdf]

lncRNA distribution on chromosome/scaffold

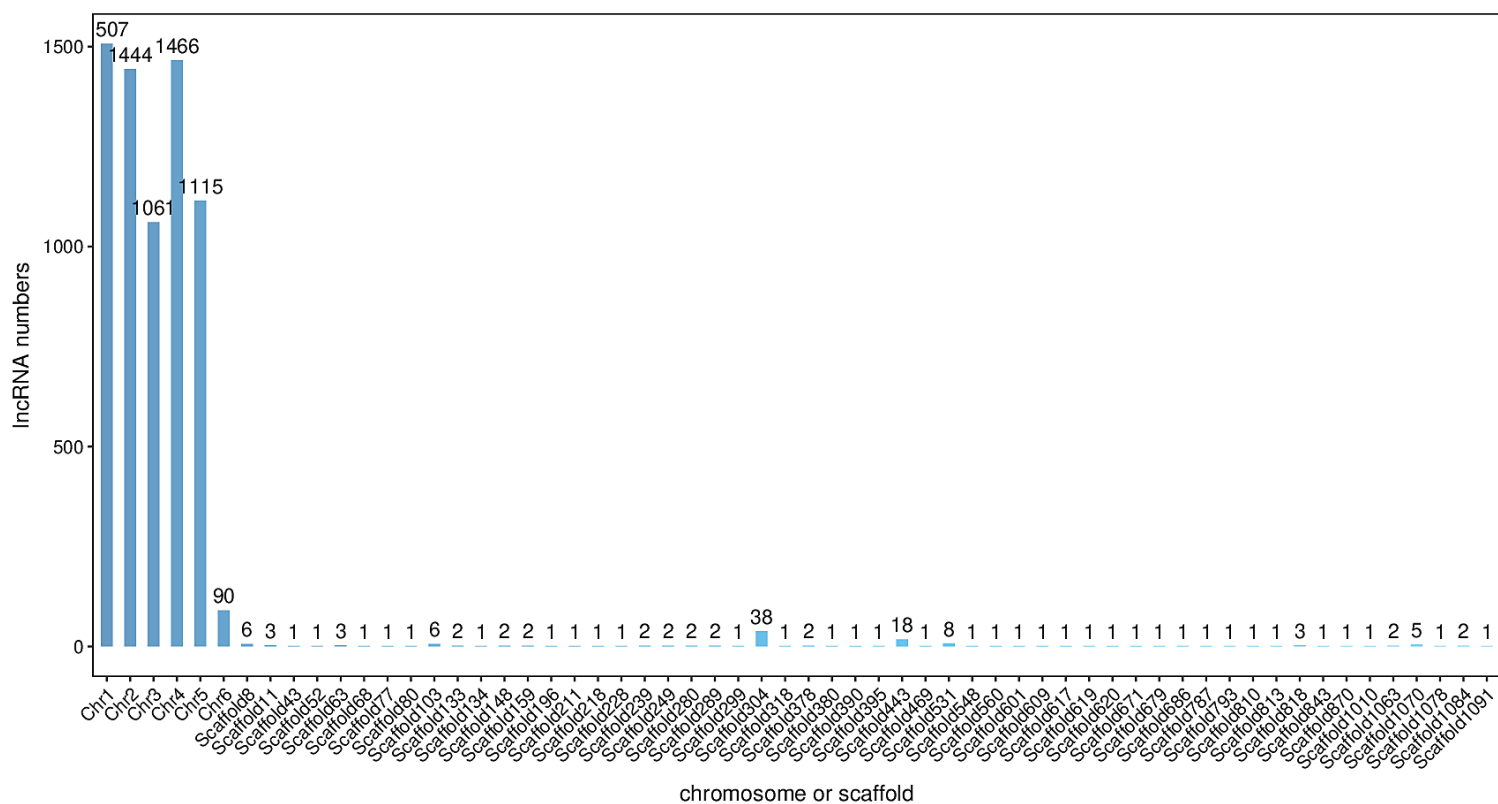

Supplement: S2 Fig — (PDF) [file pntd.0011411.s002.pdf]

A)

Boxplot for FPKM Values

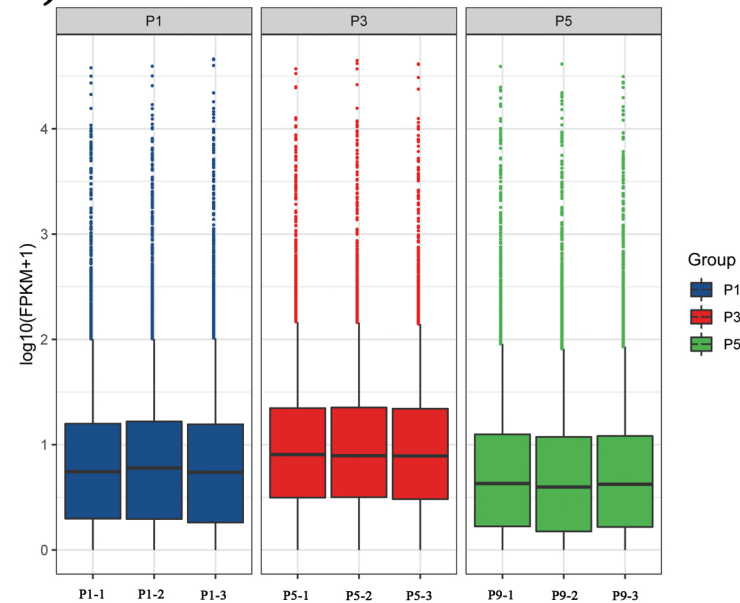

B)

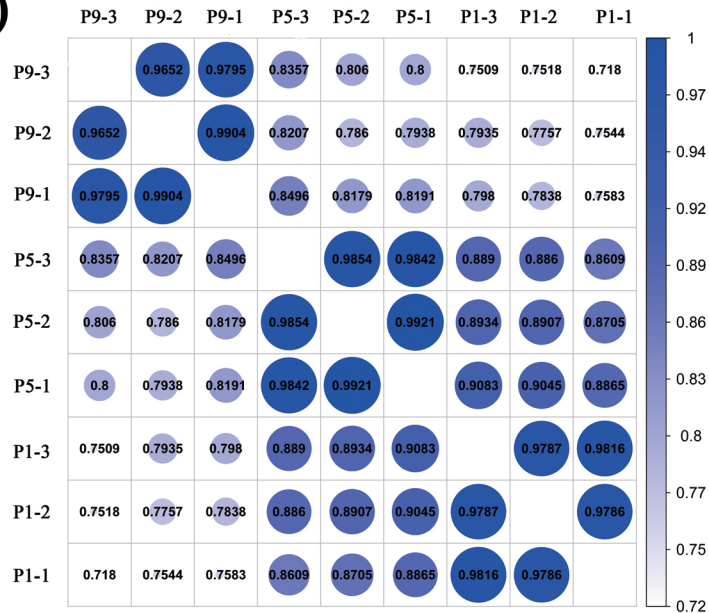

Supplement: S3 Fig — The expression level distribution of lncRNAs identified (A), the correlation coefficient between three replicate samples based on lncRNA expression (B), in pupae tissues of S. peregrina. (PDF) [file pntd.0011411.s003.pdf]

**(A)**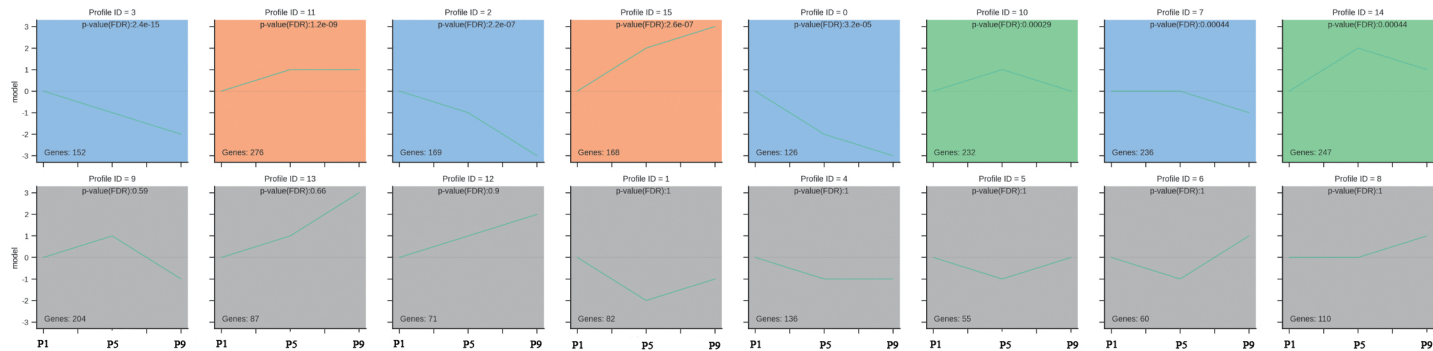**(B)**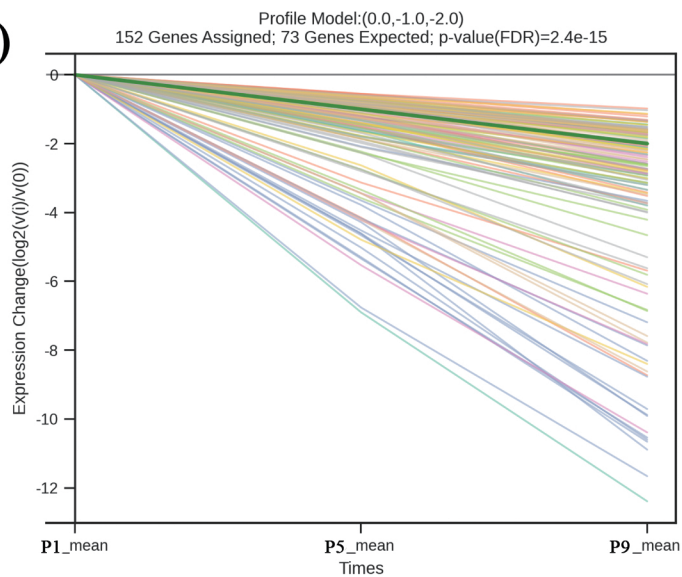**(C)**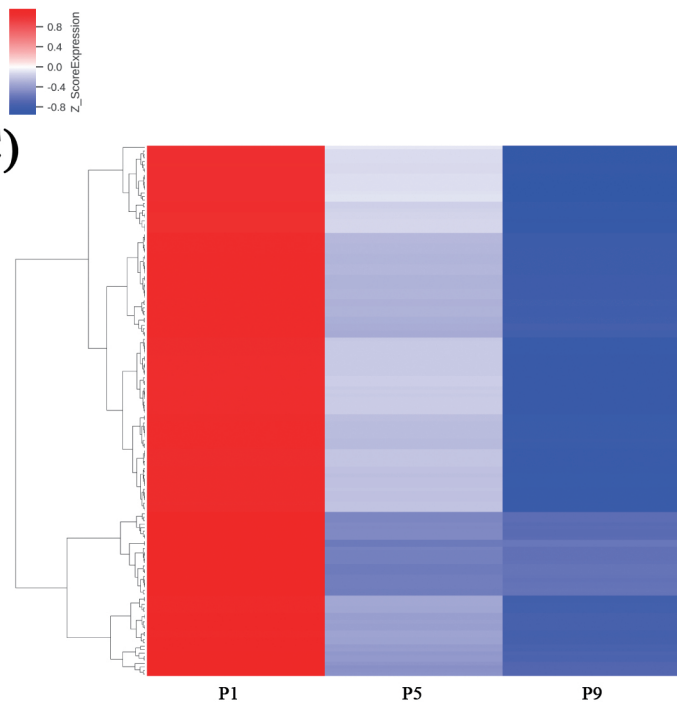

Supplement: S4 Fig — Time-series expression profiles of DE lncRNAs of S. peregrina (A); Significant trend chart (B) and cluster heat map (C) of cluster three, the y-axis indicates the expression value after homogenization, and the x-axis indicates the developmental stage, including the early pupa stage (P1), mid-term pupa stage (P5), and later pupa stage (P9). (PDF) [file pntd.0011411.s004.pdf]
