# Supplementary material for: Genome-wide analysis of long noncoding RNAs and their association in regulating the metamorphosis of the Sarcophaga peregrina (Diptera: Sarcophagidae)
Source: PLoS Negl Trop Dis. 2023 Jun 26;17(6):e0011411. doi: 10.1371/journal.pntd.0011411 (PMC10328366; doi:10.1371/journal.pntd.0011411)
Supplement: S5 Fig — (PDF) [file pntd.0011411.s005.pdf]

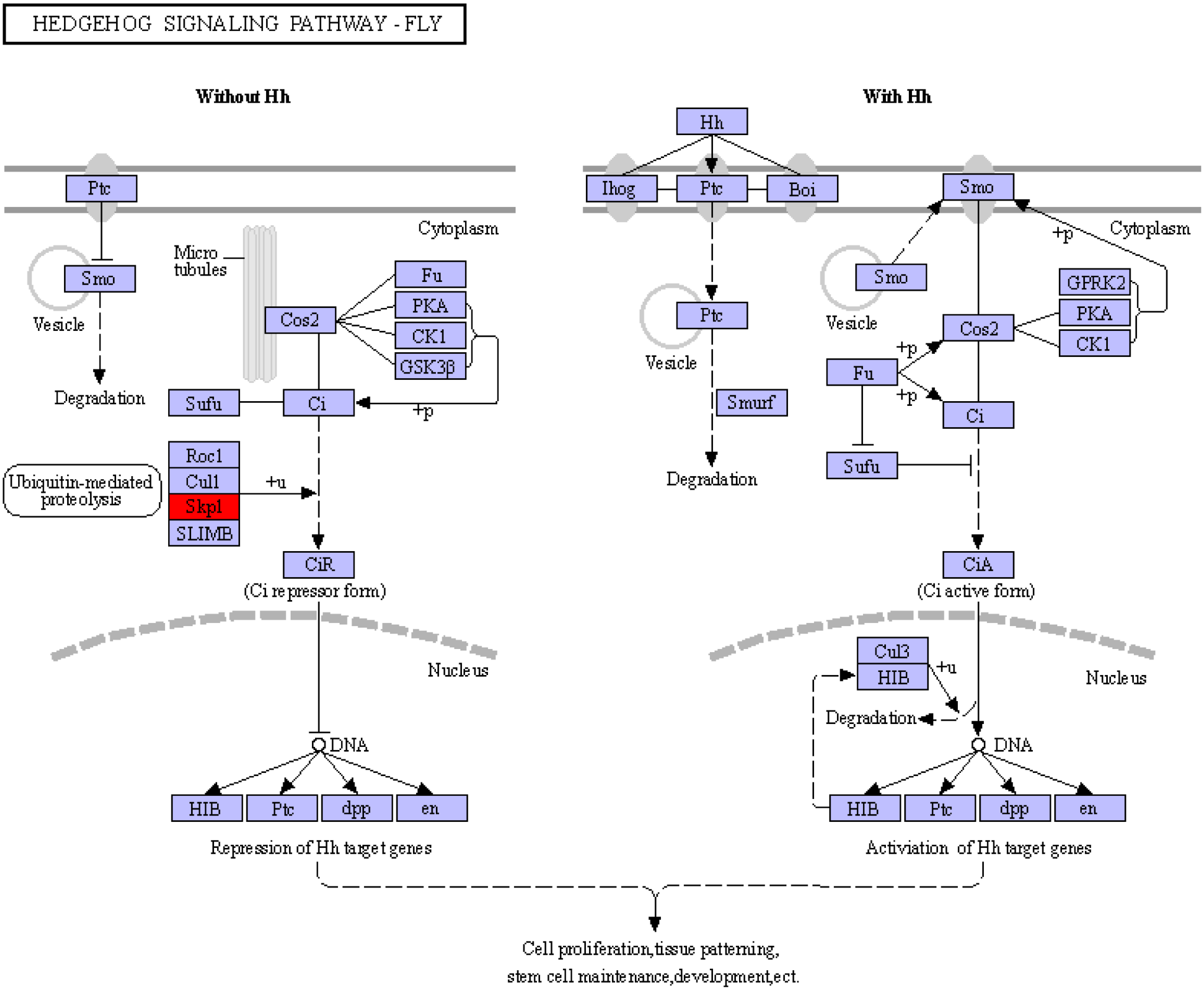
HEDGEHOG SIGNALING PATHWAY - FLY
Without Hh
With Hh
Hh
Ptc
Ihog
Boi
Smo
Cytoplasm
Micro tubules
Vesicle
Degradation
Cos2
Fu
PKA
CK1
GSK3β
GPRK2
Sufu
Ci
+p
Smurf
Roc1
Cul1
Skp1
SLIMB
Ubiquitin-mediated proteolysis
+u
CiR
(Ci repressor form)
CiA
(Ci active form)
Nucleus
Cul3
HIB
DNA
dpp
en
Repression of Hh target genes
Activiation of Hh target genes
Cell proliferation,tissue patterning,
stem cell maintenance,development,ect.
